# Novel Diacyl-hydrazide Compounds as Potential Therapeutics for Visceral Leishmaniasis

**DOI:** 10.1021/acsomega.4c04517

**Published:** 2024-08-22

**Authors:** Bernhard Jandl, Rebecca Zheng, Markus Muttenthaler, Jonathan Baell

**Affiliations:** † Institute of Biological Chemistry, Faculty of Chemistry, University of Vienna, 1090 Vienna, Austria; ‡ Vienna Doctoral School in Chemistry, University of Vienna, 1090 Vienna, Austria; § Institute for Molecular Bioscience, The University of Queensland, 4072 Brisbane, Queensland, Australia; ∥ Medicinal Chemistry, Monash Institute of Pharmaceutical Sciences, Monash University, Parkville 3052, Victoria, Australia; ⊥ School of Pharmaceutical Sciences, Nanjing Tech University, Nanjing 211816, China; # Australian Translational Medicinal Chemistry Facility, Monash University, Parkville, Victoria 3052, Australia

## Abstract

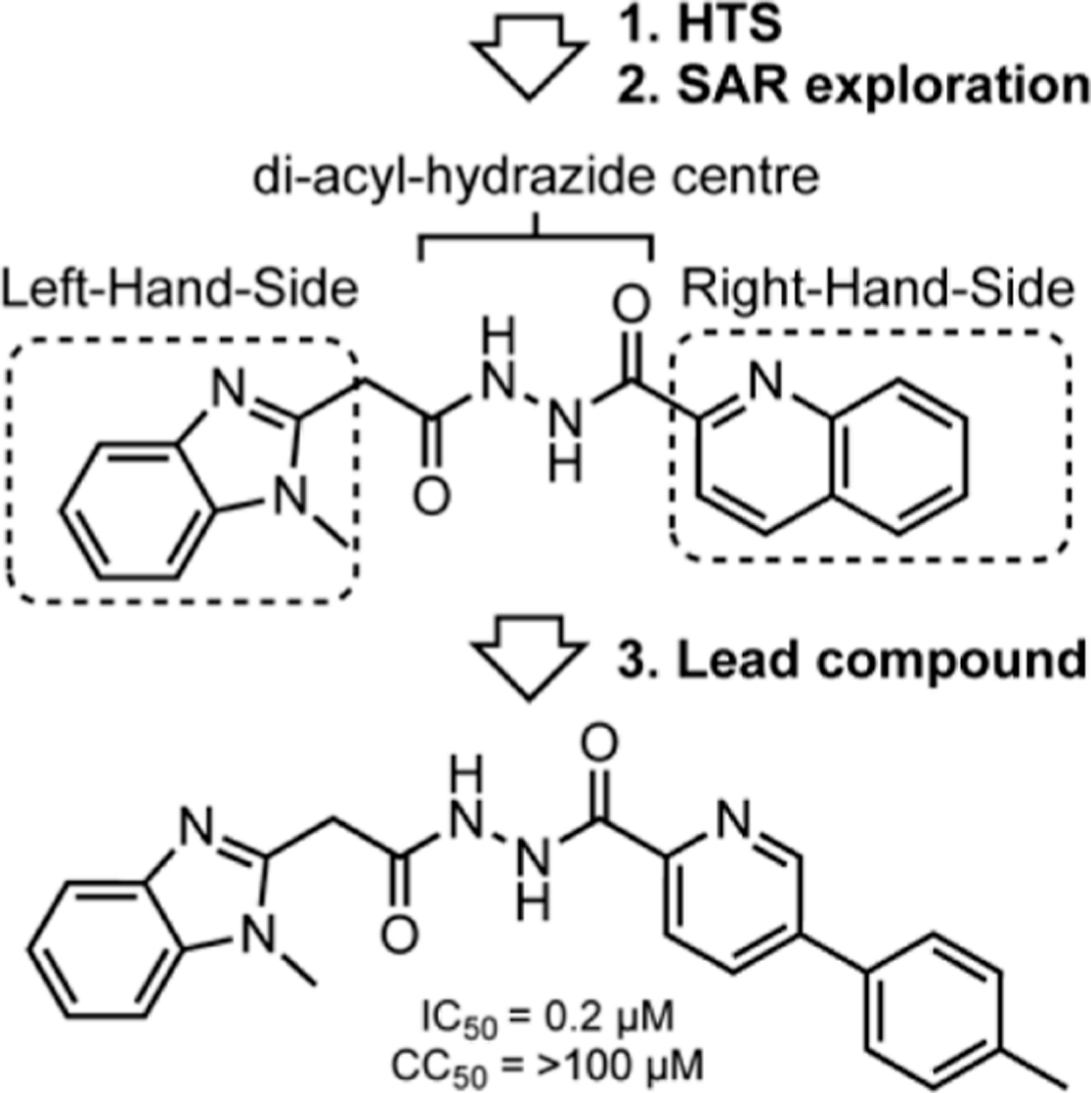

Visceral leishmaniasis is a neglected tropical disease with the highest mortality among different forms of leishmaniasis manifestation in humans. The disease is caused by the parasitic protists *Leishmania donovani* and *Leishmania infantum*, and treatments remain unsuitable due to high costs, complicated administration, lack of efficacy, variable patient susceptibility, toxic side effects, and rising parasitic resistance. Herein, we report a structure–activity relationship (SAR) exploration of the diacyl-hydrazide scaffold identified to have antiparasitic activity from a high-throughput screen against *L. donovani*, *Trypanosoma cruzi*, and *Trypanosoma brucei*. This SAR study revealed new structural insights into this scaffold related to bioactivity resulting in a new series of lead compounds with nanomolar activity against *L. donovani* and no toxicity against human THP-1 macrophages. These optimized diacyl-hydrazide compounds set the stage for future drug development and hold promise for a new treatment avenue for visceral leishmaniasis.

## Introduction

Leishmaniasis is a neglected tropical disease caused by different species of the protozoan parasite Leishmania. After malaria, leishmaniasis is the most prevalent vector-borne infectious disease in terms of mortality and total number of patients. It is estimated that more than 12 million people have an acute infection, and between 0.7–1 million new cases are reported each year.^1^ Leishmaniasis is often related to a weak immune system, malnutrition, poverty, illiteracy, and poor housing, and the disease’s therapeutic management is very expensive, with costs of up to USD 1500 per single treatment regime.^2^

The parasitic disease has three main manifestations in humans: cutaneous leishmaniasis, mucocutaneous leishmaniasis, and visceral leishmaniasis.^3^ Out of these manifestations, visceral leishmaniasis is the most severe, with 20,000–40,000 deaths reported annually.^3−7^ The two parasites *Leishmania donovani* and *Leishmania infantum* cause visceral leishmaniasis, and most cases (∼90%) occur in India, East Africa, and Brazil. The parasitic infection can target internal organs such as the liver, spleen, and bone marrow and is characterized by symptoms such as fever, weight loss, internal organ swelling, and progressive anemia. If the infection is left untreated, visceral leishmaniasis is usually fatal after two years, either directly or due to complications such as secondary infections or hemorrhage.^8,9^ Although significant efforts have been made to develop an effective vaccine for visceral leishmaniasis, none has been approved yet.^10−12^ It is estimated that leishmaniasis is endemic in at least 88 countries, making eradication almost impossible due to large parasite reservoirs, including humans, dogs, rodents, and other wild animals. That said, vector control and chemotherapy are the main methods of disease management. However, the treatment of visceral leishmaniasis is a difficult task, and treatments suffer from high costs, complicated administration, variable efficacy among different species, variable patient susceptibility, toxic side effects, and decreasing efficacy due to rising parasitic resistance.^9,13^ This renders the search for novel treatments with an improved therapeutic profile urgently needed to benefit patient health and decrease the disease mortality rate and socioeconomic burden.

Our group recently reported a successful structure–activity relationship (SAR) exploration of a hit for leishmaniasis treatment, 4-fluoro-*N*-(5-(4-methoxyphenyl)-1-methyl-1*H*-imidazole-2-yl)benzamide, that had been identified via a high-throughput screen of a 1.8 million compound library.^14^ This screen was undertaken by the Tres Cantos Open Lab Foundation, supported by GlaxoSmithKline (GSK), the Dundee Drug Discovery Unit, and the Drugs for Neglected Diseases Initiative (DNDi) against three related kinetoplastid protists, *L. donovani*, *Trypanosoma cruzi* and *Trypanosoma brucei*.^15^ The GSK high-throughput screen also tested for sterol 14α-demethylase-demethylase (CYP51) inhibition, a prominent leishmaniasis drug target,^16−18^ as well as for cytotoxic effects against HepG2, a human liver cancer cell line, which was considered in our hit selection (Table 1).

**Table 1:** Associated Data for Hit Compound 1 Discovered in the GSK Kinetoplastid High-Throughput Screen

		hit compound **1**	selection criteria
*L. donovani* growth inhibition	pIC_50_*L. donovani* in infected macrophages	5.9	>5
	pIC_50_*L. donovani* imaging: amastigote/macrophages	6.1	>5
CYP51 inhibition	pIC_50_ CYP51	4.5	<5^b^
cytotoxicity	pCC_50_ HepG2	4	<5
physicochemical properties	molecular weight [g/mol]	359	<500^a^
	cLogP	2.5	<5^a^
	polar surface area (Å^2^)	89	
	free rotatable bonds	4	
	hydrogen bond donors	2	<5^a^
	hydrogen bond acceptors	4	<10^a^
metabolic stability	half-life *t*_1/2_ [min]	80	>60
	Cl_int,in vitro_ [μg/min/mg protein]	22	<3

a Following Lipinski’s rule;^25^ CC_50_ − half maximal cytotoxic concentration; IC_50_ − half maximal inhibition concentration; Cl_int_ − intrinsic clearance.

b Compound **1** did not inhibit CYP51, which was considered positive in our hit selection criteria due to validity concerns of CYP51 as a drug target for leishmaniasis (same criteria was also applied in our previous studies with success^14,26^). These concerns derive from the negative outcomes of clinical trials for Chagas disease chemotherapy using the repurposed antifungal drugs ravuconazole and posaconazole, which are fungal CYP51 inhibitors.^27−32^

**Figure 1:**
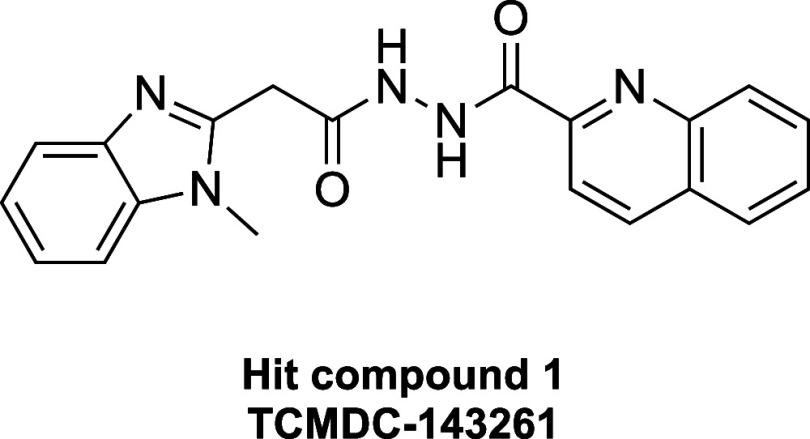
Hit compound **1** from GSK’s high-throughput screen.

This discovery screen led to three chemical boxes containing compounds with promising activity and druggability for these parasites. Further analysis of antiparasitic potency and cytotoxicity narrowed the hits to a final set of 192 compounds in the GSK Leishmaniasis Box. From this library, we selected the diacyl-hydrazide compound class (hit compound **1**, Figure 1) as the lead for this study due to its promising potency against *L. donovani* in infected macrophages (IC_50_ = ∼1.3 μM), low cytotoxicity against HepG2 cells (CC_50_ = ∼100 μM), distinct mode-of-action from CYP51 inhibition, and good therapeutic window (CC_50_/IC_50_ = ∼79). Although hydrazides and their derivatives, including the acyl-hydrazide class, are known to have various biological functions,^19−24^ the identified antiparasitic activity from the GSK screen for the diacyl-hydrazide compound **1** was novel, and no SAR exploration of hit compound **1** had been carried out. Our pursuit of a systematic SAR exploration was further supported by data from a previous antileishmanial SAR study on acyl-hydrazide derivatives that revealed potent leishmanicidal activity for benzyloxy-protected acyl-hydrazides against *Leishmania major* promastigotes^19^ and for aryl *N*-acyl-hydrazone compounds against *L. infantum*.^20^

The SAR approach to investigate compound **1** in this study examined the chemical space on the left- and right-hand side (LHS and RHS) of the central diacyl-hydrazide moiety. The structural modifications focused on alterations of the 1-methyl-1*H*-benzo[*d*]imidazole moiety on the LHS and the quinoline moiety on the RHS.

## Results and Discussion

### Physicochemical Properties of Compound **1**

Compound **1** displayed a pIC_50_ of ∼5.9 against *L. donovani* and a pCC_50_ of ∼4 against HepG2 cell lines, representing a good therapeutic window with a ∼79-fold selectivity for the parasite.^15^ We first analyzed compound **1** for physicochemical parameters such as the molecular weight, cLogP, polar surface area, freely rotatable bonds, hydrogen bond donors and acceptors and solubility at pH 2 and 6.5, as well as metabolic stability to evaluate its drug-likeness (Table 1). This analysis revealed that compound **1** adhered to Lipinski’s rule of five, and a cLogP value of 2.5 indicated good bioavailability. These “drug-like” physicochemical properties and the good synthetic accessibility of the scaffold further supported exploring the compound’s SAR to reveal valuable insights and guidance for drug developers.

### Chemistry

#### Left-Hand-Side-Modifications

**Scheme 1:**
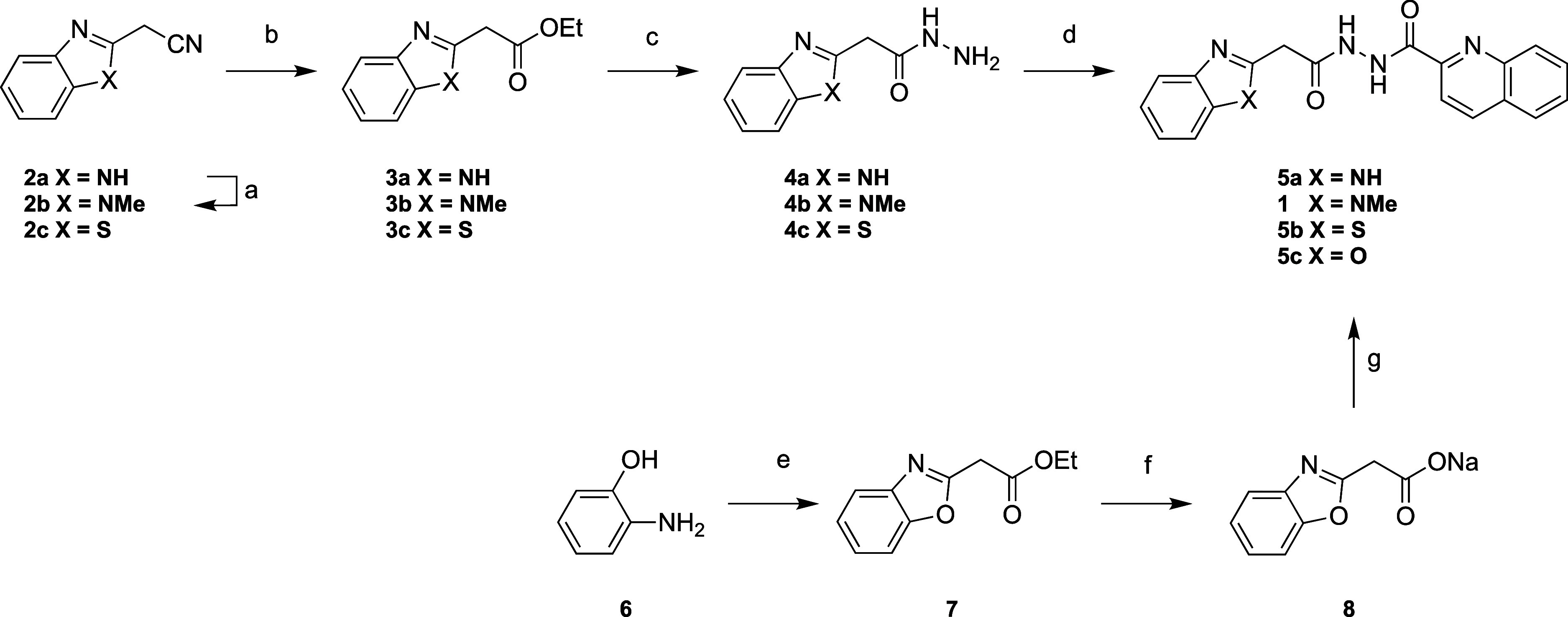
Synthetic Route to Synthesize Left-Hand-Side Modifications and Access Compounds 1 and Analogues **5a–c** Reagents and conditions: (a) dimethyl sulfate (1.2 equiv), NaOH (1.1 equiv), H_2_O, 30 °C, 83%; (b) acetyl chloride, EtOH, 80 °C, 99%, (c) hydrazine hydrate (5 equiv), EtOH, reflux, 16 h, 53–90%; (d) quinaldic acid (1 equiv), DIPEA (3 equiv), HBTU (1.3 equiv), DMF, r.t., 16 h, 32–92%; (e) ethyl 3-ethoxy-3-imino propionate HCl (1.3 equiv), EtOH, 80 °C, 16 h, 51%; (f) NaOC(CH_3_)_3_ (1 equiv), EtOH, 60 °C, 16 h, 80%; (g) quinoline-2-carbohydrazide (1.5 equiv), DIPEA (3 equiv), HBTU (1.3 equiv), DMF, r.t., 16 h, 32%.

**Scheme 2:**
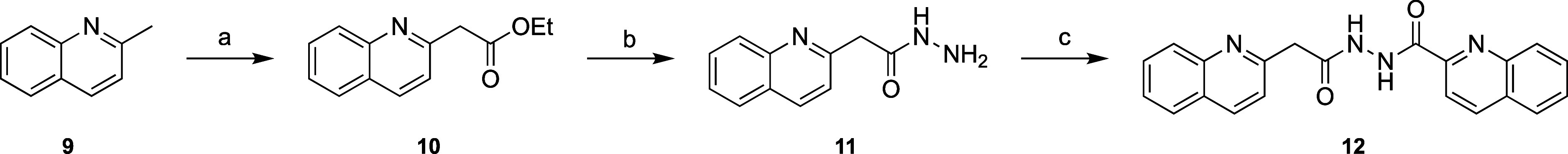
Synthetic Route to Synthesize Analogue **12** Reagents and conditions: (a) diisopropylamine (2.9 equiv), nBuLi (1.8 equiv), diethyl carbonate (3.6 equiv), THF, −78 °C, 2.5 h, 95%; (b) hydrazine hydrate (5 equiv), EtOH, reflux, 16 h, 40%; (c) quinaldic acid (1 equiv), DIPEA (3 equiv), HBTU (1.3 equiv), DMF, r.t., 16 h, 39%.

First, we synthesized analogues of compound **1** with alterations at the 1-methylbenzimidazole moiety. We used the synthetic strategy depicted in Schemes 1 and 2 to access the hit compound **1** and to generate the four related analogues **5a–c** and **12**. These analogues varied in the methylation pattern (**5a**), ring heteroatom (**5b** and **5c**) and ring size of heterocycle moiety (**12**) of the LHS imidazole scaffold. To access compound **1**, the synthetic route began with the *N*-methylation of 2-(1*H*-benzo[*d*]imidazole-2-yl)acetonitrile (**2a**) followed by the conversion to the corresponding ethyl ester (**3b**) using acetyl chloride in EtOH. This ester intermediate **3b** was then subjected to a nucleophilic substitution reaction utilizing hydrazine hydrate in EtOH to form the corresponding hydrazide (**4b**). We then used **4b** to perform an amide coupling reaction with quinaldic acid, utilizing HBTU and the non-nucleophilic base DIPEA in DMF, to yield compound **1**. A similar approach was used to synthesize the benzimidazole (**5a**) and benzothiazole analogues (**5b**). The route to synthesize analogous **5a** and **5b** began with 2-(1*H*-benzo[*d*]imidazole-2-yl)acetonitrile (**2a**) and ethyl 2-(benzo[*d*]thiazol-2-yl)acetate (**2c**), respectively, and subsequent esterification, hydrazide formation and amide coupling were carried out as outlined for compound **1**.

A different synthetic route was applied to access the benzoxazole-based analogue **5c**. This route started with the cyclization reaction between 2-aminophenol (**6**) and ethyl 3-ethoxy-3-imino propionate HCl to form compound **7**, which was then converted to the corresponding sodium salt **8** using NaOC(CH_3_)_3_ in EtOH. Then, we synthesized the final benzoxazole-based analogue **5c** via an amide coupling between the sodium salt precursor (**8**) and the quinoline-2-carbohydrazide. This approach was important in obviating the otherwise facile decarboxylation of neutralized **8**.

We additionally synthesized analogue **12** by applying a different route (Scheme 2) to install a quinoline moiety at the LHS of the diacyl-hydrazide center. In this route, we first focused on installing an ethyl ester handle to 2-methylquinoline (**9**) using LDA and diethyl carbonate to give ethyl 2-(quinolin-2-yl)acetate (**10**) in excellent 95% yield. The ester moiety of intermediate **10** was then converted to the corresponding hydrazide (**11**) using the previously described conditions to give **11**. Finally, compound **11** was reacted with quinaldic acid in an amide coupling step to obtain the desired analogue **12**.

#### Right-Hand-Side Modifications

Next, we focused on synthesizing compounds with modifications around the quinoline moiety at the RHS of the diacyl-hydrazide center of compound **1**. The symmetry of the diacyl-hydrazide center allowed us to again utilize the final amide coupling step of the above-described synthesis for LHS modifications (Scheme 1, step d) as a diversification point to access the desired RHS modifications. These modifications included analogues where the quinoline moiety was substituted by naphthyl (**13a**), pyridyl (**13b**), phenyl (**13c**), and 2- and 3-phenylpyridyl (**13d** and **13e**).

**Scheme 3:**
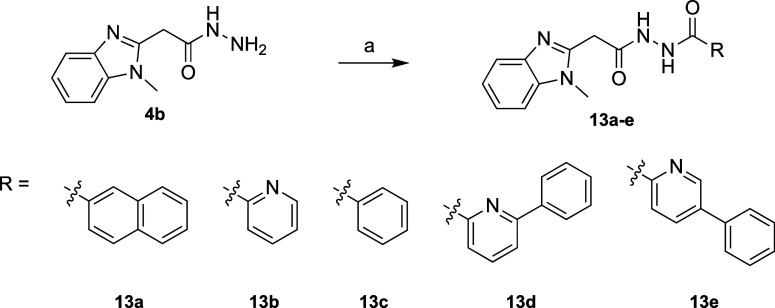
Synthetic Route to Synthesize Right-Hand-Side Modifications and Access Analogues **13a–e** Reagents and conditions: (a) DIPEA (3 equiv), HBTU (1.3 equiv), DMF, r.t., 16 h, 12–83%.

**Scheme 4:**
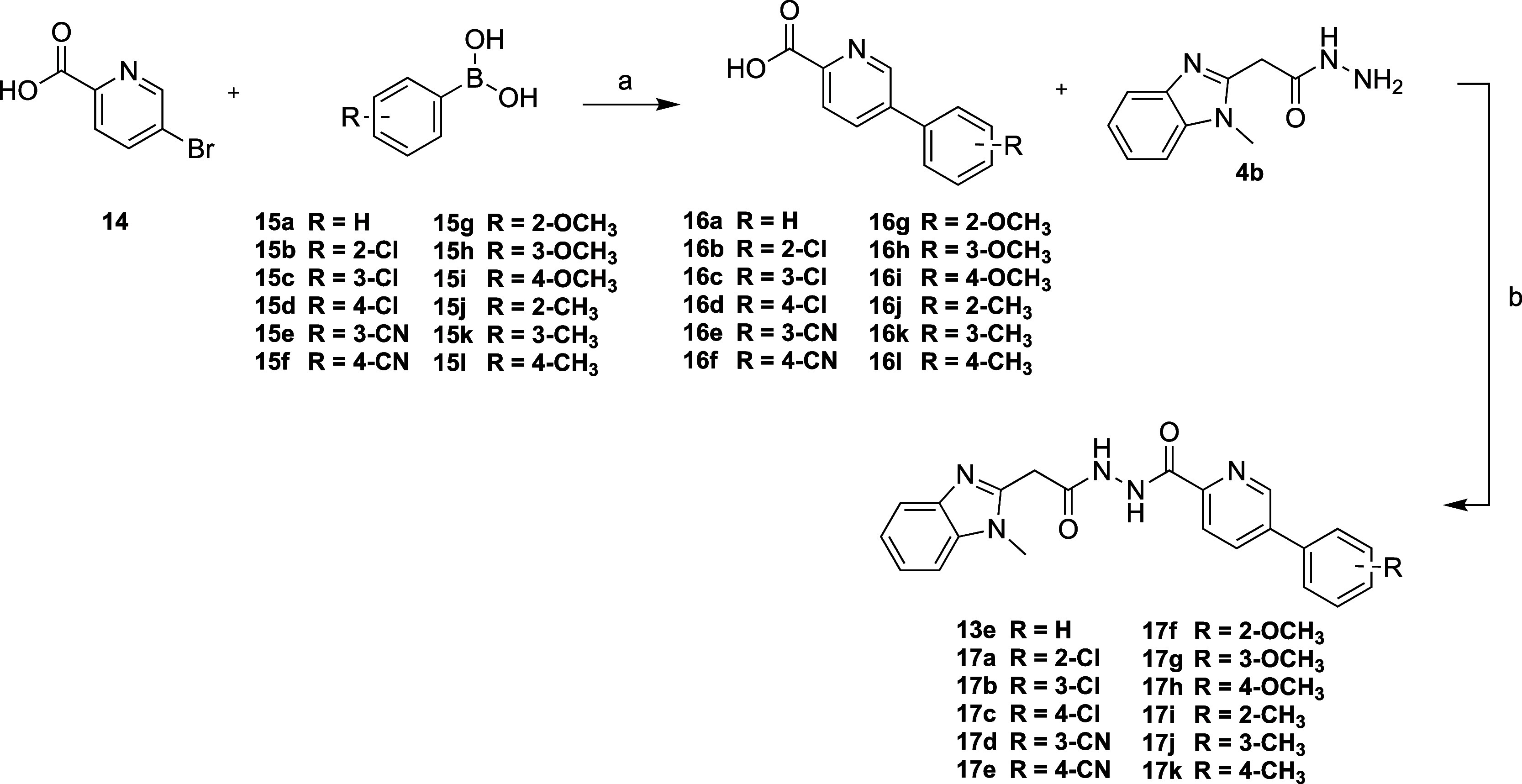
Synthetic Route to Access Analogues **13e** and **17a–k** Reagents and conditions: (a) PdCl_2_(PPh_3_)_2_, K_2_CO_3_, H_2_O, dioxane, 110 °C, 16 h, 8–42%; (b) DIPEA (3 equiv), HBTU (1.3 equiv), DMF, r.t., 16 h, 8–42%.

To access these RHS-modified analogues, we used amide couplings between **4b** and the corresponding carboxylic acid precursors (Scheme 3), yielding analogues **13a–e** between 12–83%. All carboxylic acid precursors were commercially available except for **16a**, which was used to access the final analogue **13e**. We synthesized precursor **16a** via Suzuki coupling of 5-bromopicolinic acid (**14**) with phenylboronic acid (**15a**) and subjected it to a subsequent amide coupling with **4b** to access the final analogue **13e** (Scheme 4).

Due to the promising biological results of compound **13e** from our initial SAR library screen, we decided to explore the 3-phenylpyridine RHS moiety of **13e** in more detail. We synthesized a series of derivatives with substitutions at the phenyl ring of 3-phenylpyridine. These modifications included the introduction of −Cl, –CN, –OCH_3_ and –CH_3_ at the 2-, 3- and 4-position of the aromatic ring. We used the same route as described in Scheme 4 for the synthesis of compound **13e** to access this series (**17a–k**). The synthetic route began with a Suzuki coupling to prepare the 5-bromopicolinic acid derivatives with the corresponding substitution pattern at the phenyl moiety (**16a–k**), which served as a precursor for the follow-up amide coupling with compound **4b** (Scheme 4) to form analogues **17a–k**. For the Suzuki coupling, we used commercially available phenylboronic acid derivatives with the desired functional group substitutions at the benzene ring (**15a–k**). The final amide coupling to generate the target analogues **17a–k** was low yielding (8–42%); however, enough for biological testing was isolated and no further attempt at reaction optimization was carried out.

### Biological Results

We screened all 21 synthesized compounds in vitro against obligate intracellular stages of *L. donovani* (LRC-L52) to assess the activity of our structural modifications around the initial hit compound **1**. The applied THP-1 macrophage infection assay used differentiated, nondividing human acute monocytic leukemia cells (THP-1) and THP-1 macrophage infection with *L. donovani* amastigotes was carried out as previously described.^14,33^ The antiparasitic activity and cytotoxicity were initially determined in a 384-well plate format utilizing a single-point concentration of 50 μM to identify initial hit compounds while removing others with toxic effects. Initial hits were further assessed by a 10-point curve (0.2–100 μM). The results obtained from this THP-1 macrophage infection assay were used to calculate antiparasitic IC_50_ values and CC_50_ values against macrophages.

#### Left-Hand-Side Modifications

Our LHS modifications predominantly aimed to probe the chemical space of the five-membered heterocycle of the 1-methylbenzimidazole moiety. More specifically, we used (i) compound **5a** to study the effect of the *N*-methyl group, (ii) compounds **5b** and **c** to gain insight into the displacement of the nitrogen by other heteroatoms such as sulfur and oxygen, and (iii) compound **12** to study the effect of the ring size.

This SAR study revealed that none of our newly synthesized compounds (**5a–c** and **12**) led to improved antiparasitic activity (Table 2). We investigated the role of the *N*-methyl group with compound **5a** and found that the presence of the *N*-methyl group (**1**, IC_50_ 1.9 μM) was slightly favorable over a free amine (**5a**, IC_50_ 5.9 μM) as a weak hydrogen-bond donor. Moreover, other heteroatoms such as sulfur (**5b**) and oxygen (**5c**) as substituents for the methylated nitrogen in the 1-position of the imidazole scaffold led to a >3-fold and >25-fold reduction in antiparasitic activity compared to compound **1**, respectively. Analogue **12**, synthesized to characterize the importance of the five-membered ring, also displayed reduced activity.

**Table 2:**
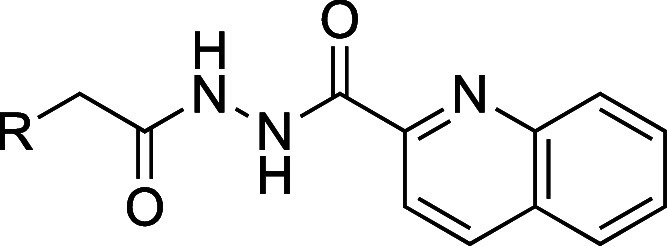

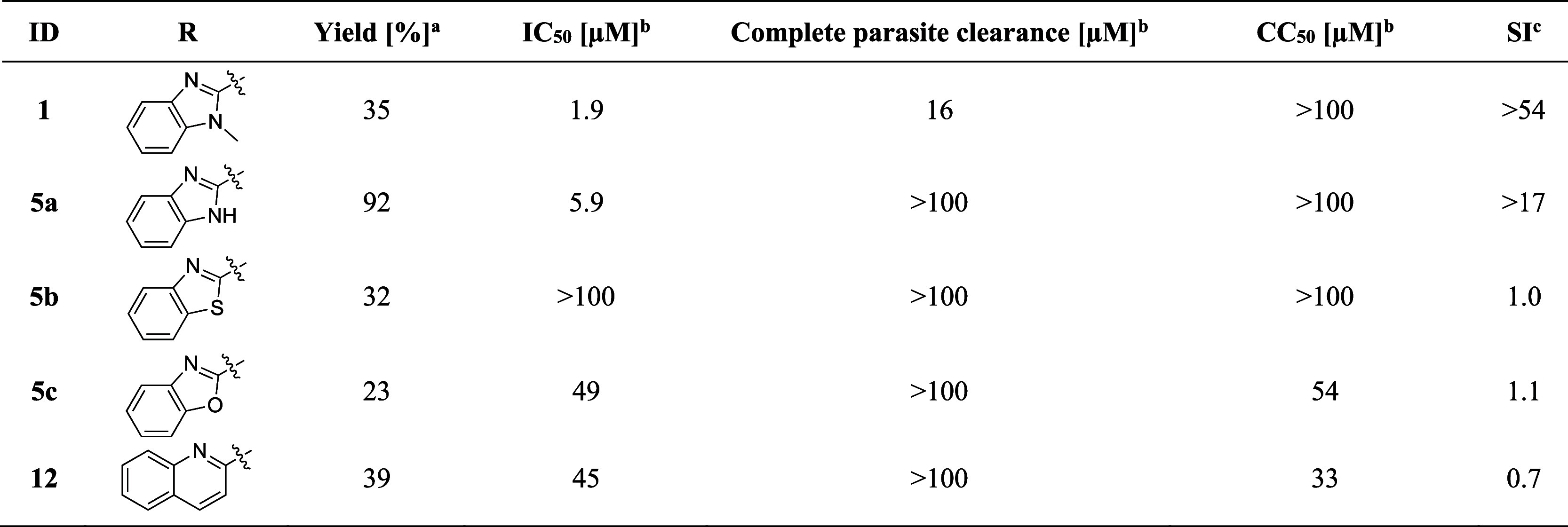
Structures, *L. donovani* Activity, Cytotoxicity and Selectivity Index (SI) with Left-Hand-Side Modification

a Yield refers to the final amide coupling step.

b Anti *L. donovani* activity and toxicity measured in THP-1 macrophage host cells using a top concentration of 100 μM (2× serial dilution 10-point curve). Experiments were performed in duplicates in one independent experiment, *n* = 1.

c SI – CC_50_/IC_50_. CC_50_ – half-maximal cytotoxic concentration. IC_50_ – half maximal inhibition concentration (reduction of total number of parasites by 50%).

Taken together, our findings underpinned that the five-membered ring structure, the presence of the nitrogen as a heteroatom, and the *N*-methyl group are LHS key features for the potent antiparasitic activity of compound **1**.

#### Right-Hand-Side Modifications

Our compound design to study the SAR of the chemical space on the RHS of the diacyl-hydrazide center focused on structural alterations of the quinoline moiety. More specifically, we assessed (i) **13a** to reveal the relevance of the nitrogen in quinoline structure, (ii) **13b** and **c** to explore the importance of the fused benzene ring to the pyridine moiety, and (iii) **13d** and **e** to shed light on a more flexible conjugation of the benzene ring in ortho- and meta-position to the pyridine nitrogen compared to the rigid fusion of the benzene ring in quinoline (Table 3).

The biological evaluation of this series (**13a–e**, Table 3) with RHS modifications revealed that analogues **13a** and **c** completely lost their *L. donovani* growth inhibitory activity, rendering the presence of nitrogen a key structural integrity for activity. The additional fused aromatic ring also positively impacted parasitic growth inhibition, and its removal (**13b**) resulted in ∼23-fold reduced activity compared to compound **1**. **13e** was the best-performing compound out of this series, revealing that conjugation of the aromatic ring at the meta-position to the nitrogen was slightly favored over the quinoline moiety of compound **1**. These results support that the aromatic moiety is important; its increased rigidity when fused, however, does not seem to be crucial for its high potency. However, it is worth mentioning that the conjugation site of the aromatic ring plays a crucial role, as compound **13d** with the aromatic ring conjugated in ortho-position to the nitrogen did not have improved activity over compound **1** and even had a >10-fold lower antiparasitic activity than the ortho-conjugated analogue **13e**.

Overall, this RHS SAR study (**13a–e**) led to the identification of compound **13e** as a strong *L. donovani* growth inhibitor with similar antiparasitic activity as the initial hit compound **1**. This result sparked our interest in conducting a more detailed SAR exploration of the newly identified 3-phenylpyridine moiety to expand on this RHS modification. Hence, we synthesized and tested an additional series to study the effects of different functional groups (−Cl, –CN, –OCH_3_ and –CH_3_) at diverse positions (2-, 3- and 4-) around the phenyl moiety (**17a–k**; Table 4). The antiparasitic screen of these analogues revealed that all compounds of this series potently inhibited *L. donovani* growth. Interestingly, independently of the functional group and position of the modification, all analogues had potent IC_50_ values ranging from 0.2–1.4 μM against the *L. donovani*. This result rendered this new compound series not only more potent than the initial hit compound **1** but also more potent than **13e**. Of note, within this compound series, 5 out of 11 had no toxic effects, and 7 out of 11 had an excellent SI of >100. **17g–k** had the best therapeutic profile, with nanomolar antiparasitic activity and no toxicity against macrophages (CC_50_ = 52 – >100 μM). **17k** (IC_50_ = 0.2 μM, CC_50_ = >100 μM, SI = >500) was the best-performing compound from this series, with ∼10-fold increased activity against *L. donovani* compared to **1**.

**Table 3:**
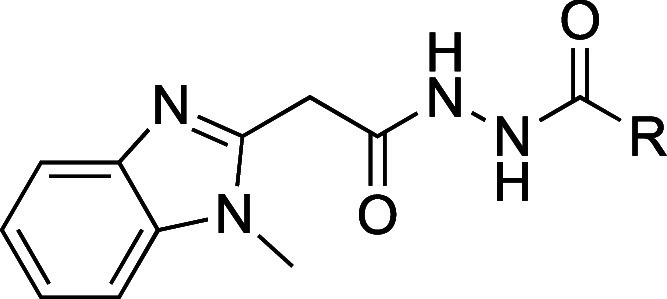

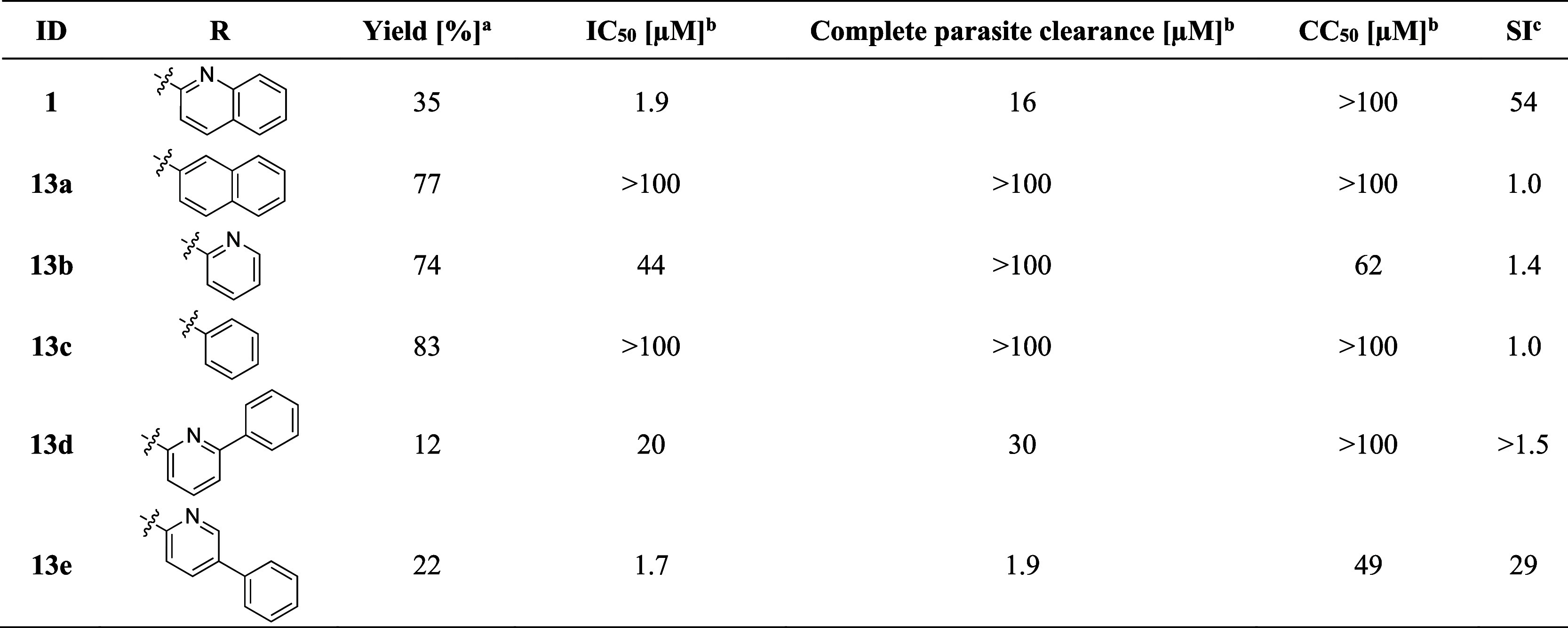
Structures, Anti *L. donovani* Activity, Cytotoxicity and SI with Right-Hand-Side Modification

a Yield refers to the final amide coupling step.

b Anti *L. donovani* activity and toxicity measured in THP-1 macrophage host cells using a top concentration of 100 μM (2× serial dilution 10-point curve). Experiments were performed in duplicates in one independent experiment, *n* = 1.

c SI—CC_50_/IC_50_. CC_50_—half-maximal cytotoxic concentration. IC_50_—half maximal inhibition concentration (reduction of total number of parasites by 50%).

## Conclusion

**Figure 2:**
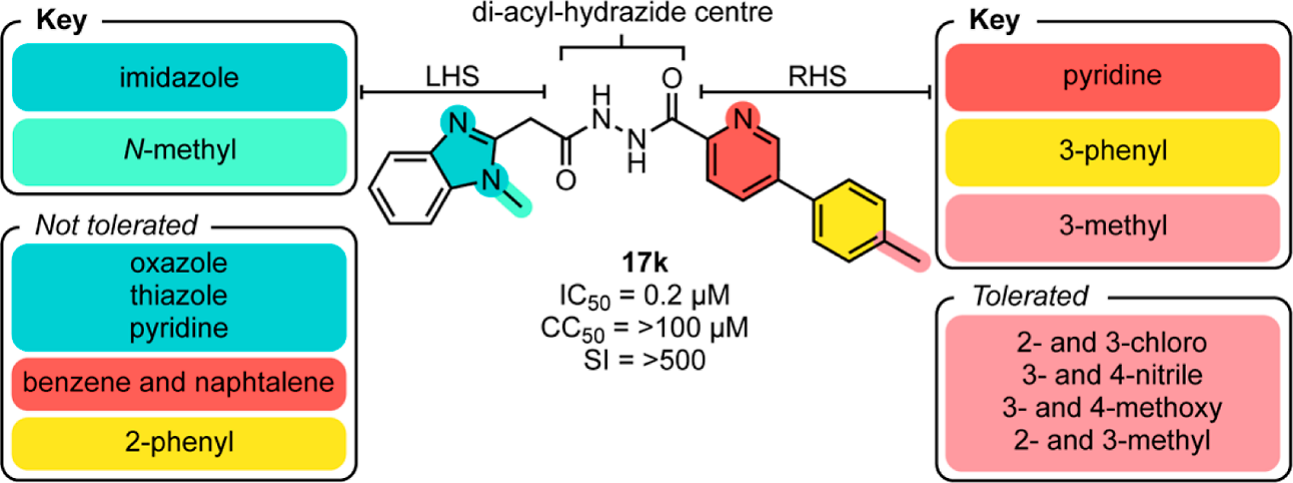
Key structural elements identified in this study.

We synthesized 20 analogues to explore the chemical space on the LHS and RHS of the diacyl-hydrazide center of initial hit compound **1**. This approach yielded several key structural insights (Figure 2) that eventually led to novel structures with improved therapeutic potential against *L. donovani* parasites (**17g–k**). Each of these leads had nanomolar to low micromolar antileishmanial activity (IC_50_ = 0.2 – 1 μM) with no cytotoxicity to human macrophages (CC_50_ = 52 – >100 μM; SI = >145 – >500). Compound **17k** was the best lead, with nanomolar activity against the parasite *L. donovani* (IC_50_ = 200 nM; CC_50_ = >100 μM; SI = >500). Potential off-target effects against human kinases, proteases, G protein-coupled receptors and cytochrome p450, however, should be profiled before pursuing these leads further. Taken together, this study opens a new avenue toward treating visceral leishmaniasis and provides a good starting point for further drug development based on the diacyl-hydrazide compound class.

## Experimental Section

### Parasite and Cell Cultures

*L. donovani* MHOM/SD/62/1S-CL2D parasites were cultured as promastigotes at 28 °C in M199 medium (Sigma-Aldrich, St. Louis, MO, USA) with 40 mM HEPES, 0.1 mM adenine, 0.0001% biotin, and 4.62 mM NaHCO_3_ supplemented with 10% fetal bovine serum (FBS, Gibco, Carlsbad, CA, USA), 100 μg/mL penicillin (Gibco), and 100 μg/mL streptomycin (Gibco). THP-1 cells (ATCC TIB-202) were cultured in RPMI-1640 medium containing 4.5 g/L glucose, 10 mM HEPES, 1 mM sodium pyruvate, and 10% FBS. The cells were maintained in tissue culture flasks (Nunc A/S, Roskilde, Denmark) in a 5% CO_2_ incubator at 37 °C. The parasites were subcultured every 3 or 4 days and were maintained for 10 passages.

### Screening of Bioactive Compounds Against Intracellular Leishmania

PMA-treated THP-1 human monocytic cells were seeded at 0.8 × 10^4^ cells per well in a 384-well culture plate (Greiner Bio-One, Kremsmünster, Austria) in RPMI-1640 complete medium supplemented with 10% FBS. After 48 h of incubation at 37 °C in the presence of 5% CO_2_, the promastigotes of *L. donovani* that were incubated with lectin for 30 min at 28 °C were added to the cells at a parasite-to-cell ratio of 20:1. Infected THP-1 cells were treated with amphotericin B (at 4 μM, positive control), miltefosine (at 10 μM, positive control), and screening compounds (at 10 μM). The negative control consisted of THP-1 infected with the parasite with only 0.5% DMSO. After 72 h, the cells that were infected and treated with the drug were washed with serum-free RPMI-1640 medium. The cells and parasites were stained using 5 μM DRAQ5 and 4% PFA. The images were acquired based on reading using an Operetta automated microscope (PerkinElmer, Inc., Waltham, MA 02451 USA). They were further analyzed using Columbus (PerkinElmer, Inc. Waltham, MA, USA) software to quantify parasite numbers, host cell numbers, and infection ratios. In brief, the large-sized nucleus of host cells was first detected using Draq-5 (Thermo Fisher, Rockford, IL, USA) signal and the host cell boundary masking was performed using the low-intensity signals from cytosols (an additional feature of Draq-5). Then, the small-sized nucleus signal by Draq-5 was used to identify parasites within the area of the masked host cell. IR was determined with the value of the number of infected cells divided by the total number of cells, and the average number of parasites per macrophage (*P*/φ) was defined by the value of the number of parasites divided by the number of infected cells in the acquired image. The average IR value of the negative control wells was calculated as 0.53. Compounds selected based on the screening results were further assessed in a dose-dilution manner (2-fold serial dilution for 10 points starting from 100 μM) using the same method.

### Parasite Growth Inhibition

*L. donovani* promastigote growth inhibition was assayed by measuring the conversion of resazurin to resorufin. The assays were performed in 384-well plates seeded with *L. donovani* promastigotes (5 × 10^4^ cells per well). After seeding, the parasites were exposed to the compounds for 3 days. Resazurin sodium salt (200 μM; R7017; Sigma-Aldrich, St. Louis, MO, USA) was added, and the samples were incubated for 5 h. After incubation, the parasites were fixed using 4% paraformaldehyde, and the plates were analyzed using a Victor3 plate reader (PerkinElmer, Inc., Waltham, MA, USA) at 590 nm (emission) and 530 nm (excitation). Amphotericin B and miltefosine were the reference drugs for the *L. donovani* promastigote growth inhibition.

### Chemistry

#### General

All solvents used were of analytical grade: ethyl acetate (EtOAc); dichloromethane (DCM); dimethylformamide (DMF); methanol (MeOH); tetrahydrofuran (THF), and ethanol (EtOH). ^1^H and ^13^C Nuclear Magnetic Resonance (NMR) spectra were recorded at 400.13 and 101 Hz, respectively, on a Bruker Avance III Nanobay 400 MHz spectrometer coupled to the BACS 60 automatic sample changer at 25 °C. Results are recorded as follows: chemical shifts (δ) in ppm acquired in either CDCl_3_ (7.26 ppm for ^1^H and 77.16 ppm for ^13^C), DMSO-*d*_6_ (2.50 ppm for ^1^H and 39.52 ppm for ^13^C) or MeOD (3.31 ppm for ^1^H and 49.00 ppm for ^13^C) as a reference. Solvents used for NMR studies are from Cambridge Isotope Laboratories. Each proton resonance was assigned according to the following convention: chemical shift (δ), multiplicity, coupling constant (*J*), expressed in hertz (Hz), and number of protons. Each carbon resonance was assigned according to the following convention: chemical shift (δ), multiplicity (where no multiplicity is assigned a singlet peak was observed). Analytical HPLC was acquired on an Agilent 1260 Infinity analytical HPLC coupled with a G1322A degasser, G1312B binary pump, G1367E high-performance autosampler, G4212B diode array detector. Conditions: Zorbax Eclipse Plus C18 Rapid resolution column (4.6 × 100 mm) with UV detection at 254 and 214 nm, 30 °C; sample was eluted using a gradient of 5–100% solvent B in solvent A where solvent A: 0.1% formic acid in water, and solvent B: 0.1% formic acid in MeCN (5 to 100% B [9 min], 100% B [1 min]; 0.5 mL/min). Low-resolution mass spectrometry (LCMS) was performed on an Agilent 6100 Series Single Quad LCMS coupled with an Agilent 1200 series HPLC, G1311A quaternary pump, G1329A thermostated autosampler, and G1314B variable wavelength detected (214 and 254 nm). LC conditions: Phenomenex Luna C8(2) column (100 Å, 5 μm, 50 × 4.6 mm), 30 °C; sample (5 μL) was eluted using a binary gradient (solvent A: 0.1% aq. HCO_2_H; solvent B: 0.1% HCO_2_H in MeCN; 5 to 100% B [10 min]; 100% B [10 min]; 0.5 mL/min). MS conditions: quadrupole ion source with multimode-ESI; drying gas temperature, 300 °C; vaporizer temperature, 200 °C; capillary voltage, 2000 V (positive mode) or 4000 V (negative mode); scan range, 100–1000 *m*/*z*; step size, 0.1 s over acquisition time 10 min. High-resolution mass spectrometry (HRMS) analysis was performed on an Agilent 6224 TOF LCMS coupled to an Agilent 1290 Infinity LC. All data were acquired, and reference mass was corrected via a dual-spray electrospray ionization (ESI) source. Acquisition and analysis were performed using the MassLynx software version 4.1 Mass Spectrometer with the following conditions: ESI mode; desolvation gas flow: 550 L/h; desolvation temperature: 250 °C; source temperature: 110 °C; capillary voltage: 2400 V; sample cone voltage: 60 V; scan range acquired: 100–1500 *m*/*z*; scan time: 1 s; internal reference ions: positive ion mode *m*/*z* = 556.2771.

#### General Procedure A—Hydrazide Handle Formation

To a solution of appropriate ethyl ester (1 mmol) in EtOH (3 mL) was added hydrazine hydrate solution (5 mmol). The reaction was refluxed overnight. Upon completion, the reaction was poured on ice, filtered, and dried via suction filtration to give the desired compound as a solid.

#### General Procedure B—Amide Coupling

To a solution of the appropriate amine (1.5 equiv) and DIPEA (3 equiv) in DMF (3 mL/mmol) was added the various carboxylic acids (1 equiv). HBTU (1.3 equiv) was added to the mixture. The solution was stirred at r.t. for 16 h. The reaction was concentrated in vacuo and washed with EtOAc and brine. If a solid was formed, the reaction was filtered via vacuum filtration and washed with EtOAc, and the product was collected as a solid. If required, further purification was conducted using column chromatography and various solvents, depending on the compound.

#### General Procedure C—Suzuki Coupling

To a solution of 5-bromopicolinic acid (1 mmol, 202 mg), appropriate boronic acid (1.3 eq, 1.3 mmol), and K_2_CO_3_ (1.8 eq, 1.8 mmol) in dioxane/water (30 mL, v/v, 3/1) was added Pd(dppf)Cl_2_ (0.03 eq, 0.03 mmol). The reaction mixture was stirred at 110 °C under N_2_ overnight. The reaction mixture was cooled to room temperature, and the pH was adjusted to 9–10. The mixture was filtered through Celite. The aqueous layer was washed with Et_2_O. The aqueous layer was collected, and the pH was adjusted to ∼4–5 with 1 N HCl and extracted with EtOAc. The organic layers were collected, dried over MgSO_4_ and concentrated in vacuo to produce the desired product.

#### 2-(1-Methyl-1*H*-benzo[d]imidazole-2-yl)acetonitrile (**2b**)

To a solution of 1*H*-benzimidazole-2-acetonitrile (1.0 g, 6.4 mmol) and NaOH (1.1 equiv) in water (5 mL) was added dimethyl sulfate (1.2 equiv) dropwise. The mixture was heated to 30 °C for 1 h. Upon reaction completion, the mixture was cooled, and the precipitate formed was filtered via suction filtration, washed with water, dried and collected. Compound **2b** was obtained as a light brown solid (710 mg, 83%). HPLC – *t*_R_ = 2.26 min, >99% purity at 254 nm; LRMS [M + H]^+^ 172.0 *m*/*z*; ^1^H NMR (400 MHz, DMSO-*d*_6_): δ = 7.64–7.60 (m, 1H), 7.57–7.51 (m, 1H), 7.30–7.17 (m, 2H), 4.52 (s, 2H), 3.75 (s, 3H); ^13^C NMR (101 MHz, DMSO-*d*_6_): δ = 145.7, 141.7, 135.9, 122.5, 121.9, 118.9, 116.3, 110.2, 29.8, 17.4 ppm.

#### Ethyl 2-(1-Methyl-1*H*-benzo[*d*]imidazole-2-yl)acetate (**3b**)

To a solution of 2-(1-Methyl-1*H*-benzo[*d*]8midazole-2-yl) acetonitrile (171 mg, 1 mmol) in EtOH (10 mL) was added acetyl chloride (2.5 mL, 15 mmol) dropwise at 0 °C. The mixture was heated to reflux for 2 h, cooled to room temperature and concentrated in vacuo. The hydrochloric salt was dissolved in water and neutralized with a NaHCO_3_ solution. The solution was extracted with DCM, and the organic layers were collected and dried over MgSO_4_, filtered and concentrated to give compound **3b** as a brown oil (217 mg, 99%). HPLC – *t*_R_ = 3.32 min, >99% purity at 254 nm; LRMS [M + H]^+^ 229.0 *m*/*z*; ^1^H NMR (400 MHz, MeOD): δ = 7.64–7.58 (m, 1H), 7.53–7.48 (m, 1H), 7.35–7.25 (m, 2H), 4.23 (q, *J* = 7.1 Hz, 2H), 3.83 (s, 3H), 1.28 ppm (t, *J* = 7.1 Hz, 3H); ^13^C NMR (101 MHz, DMSO-*d*_6_): δ = 145.6, 141.7, 135.9, 135.8, 122.4, 121.8, 118.8, 116.2, 110.1, 60.9, 33.7, 29.8, 17.4 ppm.

#### 2-(1-Methyl-1*H*-benzo[*d*]imidazole-2-yl)acetohydrazide (**4b**)

Compound **4b** was prepared from ethyl 2-(1-methyl-1*H*-benzo[*d*]8midazole-2-yl) acetate (218 mg, 1 mmol) according to General Procedure A as a white solid (118 mg, 90%). LRMS [M + H]^+^ 205.1 *m*/*z*; ^1^H NMR (400 MHz, MeOD): δH 7.62–7.58 (m, 1H), 7.51–7.48 (m, 1H), 7.33–7.23 (m, 2H), 3.93 (s, 2H), 3.85 ppm (s, 3H); ^13^C NMR (101 MHz, DMSO-*d*_6_): δ = 166.9, 150.4, 141.7, 136.1, 122.5, 122.1, 118.6, 110.5, 33.4, 30.6 ppm.

#### 2-(Benzo[*d*]thiazol-2-yl)acetohydrazide (**4c**)

Compound **4c** was prepared from ethyl 2-(benzo[*d*]thiazol-2-yl) acetate (224 mg, 1 mmol) according to General Procedure A as a white powder (109 mg, 53%). HPLC – *t*_R_ = 4.12 min, >99% purity at 254 nm; LRMS [M + H]^+^ 208.0 *m*/*z*; ^1^H NMR (400 MHz, DMSO-*d*_6_): δ = 9.45 (s, 1H), 8.06 (d, *J* = 7.7 Hz, 1H), 7.94 (d, *J* = 8.1 Hz, 1H), 7.51–7.46 (m, 1H), 7.44–7.39 (m, 1H), 4.35 (s, 2H), 3.97 ppm (s, 1H); ^13^C NMR (101 MHz, DMSO-*d*_6_): δ = 166.5, 165.2, 152.2, 130.3, 126.0, 124.9, 122.2, 121.9, 36.8 ppm.

#### Ethyl 2-(1*H*-Benzo[d]imidazole-2-yl)acetate (**3a**)

A solution of 1*H*-benzimidazole-2-acetonitrile (400 mg, 2.5 mmol) in EtOH (5 mL) was cooled to 0 °C and added acetyl chloride (5 mL) dropwise. The reaction was refluxed until completion. Upon completion, the reaction was cooled and concentrated in vacuo, and the HCl salt was neutralized by NaHCO_3_. The reaction was extracted with DCM. The organic layers were collected, dried over MgSO_4_, filtered and dried under reduced pressure to give the compound **3a** as a brown oil (445 mg, 89%). HPLC – *t*_R_ = 3.05 min, > 90% purity at 254 nm; LRMS [M + H]^+^ 205.1 *m*/*z*; ^1^H NMR (400 MHz, MeOD): δH 7.53 (s, 2H), 7.29–2.17 (m. 2H), 5.49 (s, 2H), 4.22 (q, *J* = 7.1 Hz, 2H), 1.27 ppm (t, *J* = 7.1 Hz, 3H). ^13^C NMR (101 MHz, DMSO-*d*_6_): δ = 168.7, 147.7, 121.9, 121.0, 118.4, 111.1, 60.8, 35.1, 14.0 ppm.

#### 2-(1*H*-Benzo[*d*]imidazole-2-yl)acetohydrazide (**4a**)

Compound **4a** was prepared from ethyl 2-(1*H*-benzo[*d*]imidazole-2-yl)acetate (200 mg, 1 mmol) according to General Procedure A as a white solid (149 mg, 78%). HPLC – *t*_R_ = 1.504 min, >95% purity at 254 nm; LRMS [M + H]^+^ 191.1 *m*/*z*; ^1^H NMR (400 MHz, DMSO-*d*_6_): δ = 9.27 (s, 1H), 7.47–7.27 (m, 2H), 7.02 (q, *J* = 4.3 Hz, 2H), 4.20 (s, 2H), 2.58 ppm (s, 2H).

#### Ethyl 2-(Imidazole-2-yl)acetate (**10**)

A mixture of DIPA (1.46 g, 14.5 mmol) and dried THF (2.2 mL) was added to a flask under N_2_ and cooled to −78 °C. Once cooled, *n*-BuLi (0.58 g, 9 mmol) was slowly added, and the reaction was stirred for 30 min. 2-Methylquinoline (0.71 g, 5 mmol) was added and stirred for 30 min before adding diethyl carbonate (2.21 g, 18 mmol). The reaction was stirred for 2 h. The reaction is diluted with water and extracted with EtOAc. The organic layer was collected, dried over MgSO_4_, filtered and concentrated in vacuo to give the compound **10**. HPLC – *t*_R_ = 3.25 4 min, >50% purity at 254 nm; LRMS [M + H]^+^ 216.1 *m*/*z*; ^1^H NMR (400 MHz, DMSO-*d*_6_): δ = 8.33 (d, *J* = 8.4, 1H), 8.00–7.93 (m, 2H), 7.74 (ddd, *J* = 8.3, 6.9, 1.5 Hz, 1H), 7.58 (ddd, *J* = 8.2, 6.9, 1.2 Hz, 1H), 7.51 (d, *J* = 8.4 Hz, 1H), 4.12 (q, *J* = 7.1 Hz, 2H), 4.04 (s, 2H), 1.19 ppm (t, *J* = 7.1 Hz, 3H).

#### 2-(Imidazole-2-yl)acetohydrazide (**11**)

Compound **11** was prepared from ethyl 2-(imidazole-2-yl)acetate (200 mg, 1 mmol) according to General Procedure A as a white solid (76 mg, 40%). HPLC – *t*_R_ = 1.747 min, >95% purity at 254 nm; LRMS [M + H]^+^ 202.1 *m*/*z*; ^1^H NMR (400 MHz, DMSO): δH 9.35 (s, 1H), 8.29 (d, *J* = 8.4 Hz, 1H), 7.98–7.90 (m, 2H), 7.73 (ddd, *J* = 8.4, 6.9, 1.5 Hz, 1H), 7.57 (ddd, *J* = 8.0, 6.9, 1.2 Hz, 1H), 7.51 (d, *J* = 8.5 Hz, 1H), 4.27 (s, 2H), 3.75 ppm (s, 2H); ^13^C NMR (400 MHz, DMSO-*d*_6_): δC 168.3, 156.9, 147.1, 136.1 ppm.

#### Sodium 2-(Benzo[*d*]oxazol-2-yl)acetate (**8**)

To a solution of ethyl 2-(benzo[*d*]oxazol-2-yl)acetate (450 mg, 2.2 mmol) in water (0.03 mL, 2.2 mmol) and ethanol (15 mL) was added sodium *tert*-butoxide (210 mg, 2.2 mmol) dropwise over 30 min. The reaction was stirred at 60 °C overnight. The reaction was concentrated, and the solid was washed with diethyl ether and ethanol. The solid was dried via suction filtration and collected. Compound **8** was collected as a pale pink solid (347 mg, 80%). HPLC – *t*_R_ = 3.435min, >95% purity; LRMS [M + H]^+^ 178.0 *m*/*z*; ^1^H NMR (400 MHz, DMSO-*d*_6_): δ = ; 7.64–7.58 (m, 2H), 7.31–7.27 (m, 2H), 3.56 ppm (s, 2H).

#### Ethyl 2-(Benzo[*d*]oxazol-2-yl)acetate (**7**)

To a solution of 2-aminophenol (100 mg, 9 mmol) in ethanol was added ethyl 3-ethoxy-3-imino propionate HCl (1.3 eq, 12 mmol). The reaction mixture was stirred at 80 °C for 16 h. The reaction was concentrated in vitro and extracted with EtOAc. The organic layer was collected, dried over MgSO_4_, filtered and dried in vacuo. The crude compound was purified via silica chromatography, eluting 10% MeOH: DCM to produce compound **7** as a white solid (334 mg, 51%). HPLC – *t*_R_ = 4.451 min, >97% purity; LRMS [M + H]^+^ 206.1 *m*/*z*; ^1^H NMR (400 MHz, MeOD): δH; 7.70–7.65 (m, 1H), 7.61–7.57 (m, 1H), 7.41–7.35 (m, 2H), 4.84 (s, 2H),. 422 (q, *J* = 7.1 Hz, 2H), 1.29–1.24 ppm (m, 3H).

#### *N*′-(2-(1-Methyl-1*H*-benzo[d]imidazole-2-yl)acetyl)quinoline-2-carbohydrazide (**1**)

Compound **1** was prepared from 2-(1-methyl-1*H*-benzo[*d*]imidazole-2-yl)acetohydrazide (60 mg, 0.3 mmol) and quinaldic acid (35 mg, 0.2 mmol) according to General Procedure B, affording the compound **1** as a white solid (25 mg, 35%). HPLC – *t*_R_ = 4.12 min, >99% purity at 254 nm; HRMS (ESI) [M + H]^+^ 359.1387 *m*/*z*; found 360.1459 *m*/*z*; ^1^H NMR (400 MHz, DMSO-*d*_6_): δ = 10.78 (s, 1H), 10.54 (s, 1H), 8.59 (d, *J* = 8.5 Hz, 1H), 8.18–8.06 (m, 3H), 7.89 (t, *J* = 7.4, 1H), 7.74 (t, *J* = 7.4 Hz, 1H), 7.58 (d, *J* = 7.5 Hz, 1H), 7.53 (d, *J* = 7.5 Hz, 1H), 7.23–7.11 (m, 2H), 4.06 ppm (s, 2H), 3.81 (s, 3H); ^13^C NMR (101 MHz, DMSO-*d*_6_): δ = 166.3, 163.1, 149.5, 149.1, 146.1, 142.0, 138.1, 136.0, 130.8, 129.3, 129.0, 128.5, 128.2, 121.9, 121.5, 118.9, 118.5, 110.0, 33.3, 30.1 ppm.

#### *N*′-(2-(Benzo[*d*]thiazol-2-yl)quinoline-2-carbohydrazide (**5b**)

Compound **5b** was obtained using ethyl 2-(benzo[*d*]thiazol-2-yl)acetate (200 mg, 0.96 mmol) and quinaldic acid (130 mg, 0.74 mmol) following General Procedure B, affording compound **5b** as a white solid (86 ng, 32%). HPLC – *t*_R_ = 4.88 min, >99% purity at 254 nm; HRMS (ESI) [M + H]^+^ 362.0843 *m*/*z*; found 362.0915 *m*/*z*; ^1^H NMR (400 MHz, DMSO-*d*_6_): δ = 10.82 (s, 1H), 10.63 (s, 1H), 8.61 (d, *J* = 8.5, 1H), 8.17–8.09 (m, 4H), 7.99 (d, *J* = 8.1, 1H), 7.90 (t, *J* = 8.3, 1H), 7.75 (t, *J* = 7.3, 1H), 7.52 (t, *J* = 7.6 Hz, 1H), 7.44 (t, *J* = 8.1 Hz, 1H), 4.24 ppm (s, 2H); ^13^C NMR (101 MHz, DMSO-*d*_6_): δ = 166.3, 164.5, 162.9, 152.2, 149.1, 146.0, 137.9, 135.4, 130.7, 129.2, 128.9, 129.4, 128.1, 126.0, 124.9, 122.3, 122.0, 118.8, 38.8 ppm.

#### *N*′-(2-(1*H*-Benzo[*d*]imidazole-2-yl)acetyl)quinoline-2-carbohydrazide (**5a**)

Compound **5a** was obtained using 2-(1*H*-benzo[*d*]imidazole-2-yl)acetohydrazide (60 mg, 0.3 mmol) and quinaldic acid (36 mg, 0.2 mmol) following General Procedure B, affording compound **5a** as a brown solid (56 mg, 92%). HPLC – *t*_R_ = 4.099 min, >99% purity at 254 nm; LRMS [M + H]^+^ 346.1 *m*/*z*; ^1^H NMR (400 MHz, DMSO-*d*_6_): δ = 10.75 (s, 1H), 8.59 (d, *J* = 8.5 Hz, 1H), 8.16–8.09 (m, 3H), 7.89 (t, *J* = 7.6 Hz, 1H), 7.74 (t, *J* = 7.5 Hz, 1H), 7.52 (s, 2H), 7.16–7.14 (m, 2H), 3.92 ppm (s, 2H); ^13^C NMR (101 MHz, DMSO-*d*_6_): δ = 166.6, 163.0, 149.1, 148.7, 146.1, 138.1, 130.8, 129.3, 129.0, 128.5, 128.2, 118.8, 34.6 ppm.

#### *N*′-(2-(Benzo[*d*]oxazol-2-yl)acetyl)quinoline-2-carbohydrazide (**5c**)

Compound **5c** was obtained using 2-(benzo[*d*]oxazol-2-yl)acetohydrazide (60 mg, 0.2 mmol) and quinoline-2-carbohydrazide (60 mg, 0.3 mmol) following General Procedure B, affording compound **5c** as a white solid (16 mg, 23%). HPLC – *t*_R_ = 4.609, >99% purity at 254 nm; LRMS [M + H]^+^ 347.1 *m*/*z*; HRMS (ESI) [M + H]^+^ 347.1139 *m*/*z*; found 347.1146 *m*/*z*; ^1^H NMR (400 MHz, DMSO-*d*_6_): δ = ; 10.80 (s, 1H), 10.62 (s, 1H), 8.74–8.50 (m, 1H), 8.22–8.05 (m, 3H), 7.89 (ttt, *J* = 8.4, 5.0, 1.5 Hz, 1H), 7.78–7.59 (m, 3H), 7.52–7.28 (m. 2H), 4.08 ppm (s, 2H); ^13^C NMR (101 MHz, DMSO-*d*_6_): δ = 164.8, 162.9, 161.0, 150.5, 149.0, 147.1, 146.0, 138.0, 130.7, 129.3, 128.9, 128.4, 128.1, 125.1, 124.5, 119.5, 118.8, 110.7, 34.3 ppm.

#### *N*′-(2-(Imidazole-2-yl)acetyl)quinoline-2-carbohydrazide (**12**)

Compound **12** was obtained using 2-(imidazole-2-yl)acetohydrazide (60 mg, 0.3 mmol) and quinaldic acid (35 mg, 0.2 mmol) following General Procedure B, affording the Compound **12** as a white solid (24 mg, 39%). HPLC – *t*_R_ = 4.082, >99% purity at 254 nm; LRMS [M + H]^+^ 357.2 *m*/*z*; HRMS (ESI) [M + H]^+^ 357.1346 *m*/*z*; found 357.1352 *m*/*z*; ^1^H NMR (400 MHz, DMSO-*d*_6_): δ = ; 10.72 (s, 1H), 10.56 (s, 1H), 8.59 (d, *J* = 8.5 Hz, 1H), 8.35 (d, *J* = 8.5 Hz, 1H), 8.17–8.07 (m, 3H), 8.03–7.94 (m, 2H), 7.89 (ddd, *J* = 8.4, 6.9, 1.4 Hz, 1H), 7.75 (dddd, *J* = 9.3, 8.1 6.9, 1.4, 2H), 7.65 (d, *J* = 8.4 Hz, 1H), 7.59 (ddd, *J* = 8.1, 6.9, 1.2 Hz, 1H), 3.99 ppm (s, 2H); ^13^C NMR (101 MHz, DMSO-*d*_6_): δ = 168.1, 163.0, 156.4, 149.2, 147.1, 146.0, 138.0, 136.3, 130.7, 129.6, 129.3, 128.9, 128.4 (2C), 128.1, 127.8, 126.7, 126.2, 122.2, 118.8, 43.6 ppm.

#### 5-Phenylpicolinic Acid (**16a**)

A mixture of 5-bromo-pyridine-2-carboxylic acid (500 mg, 2.4 mmol), phenylboronic acid (315 mg, 3.1 mmol), K_2_CO_3_ (500 mg 4.8 mmol), Pd(dppf)Cl_2_ (45 mg, 0.07 mmol) in dioxane/water (30 mL, v/v, 3/1) was stirred at 110 °C under N_2_ over 16 h. The reaction mixture was cooled to room temperature, adjusted to pH ∼ 9–10 and filtered through Celite. The aqueous layer was washed with Et_2_O. The separated aqueous layer was adjusted to pH ∼ 4–5 with 1 N HCl and extracted with EtOAc. The combined organic layer was dried over MgSO_4_ and concentrated to give the compound **16a** as a white solid (196 mg, 50%). HPLC – *t*_R_ = 3.438 min, >95% purity; LRMS [M + H]^+^ 200.1 *m*/*z*; ^1^H NMR (400 MHz, DMSO-*d*_6_): δ = ; 9.02 (dd, *J* = 2.3, 0.8 Hz, 1H), 8.26 (dd, *J* = 8.2, 2.4 Hz, 1H), 8.12 (dd, *J* = 8.2, 0.8 Hz, 1H), 7.85–7.79 (m, 2H), 7.57–7.51 (m, 2H), 7.51–7.46 ppm (m, 1H); ^13^C NMR (101 MHz, DMSO-*d*_6_) 166.0, 150.5, 147.5, 147.0, 138.3, 136.0, 135.1, 129.2, 128.9, 127.2, 124.8 ppm.

#### 5-(2-Chlorophenyl)picolinic Acid (**16b**)

Compound **16b** was prepared using 5-bromopicolinic acid (1 mmol, 202 mg) and 2-chlorophenyl boronic acid (1.3 eq, 203 mg) according to General Procedure C, affording compound **16b** as an off-white solid (151 mg, 65%); HPLC – *t*_R_ = 3.749 min, >95% purity; LRMS [M + H]^+^ 234.0 *m*/*z*; ^1^H NMR (400 MHz, DMSO-*d*_6_): δ = ; 8.77 (d, *J* = 2.1 Hz, 1H), 8.14 (d, *J* = 8.0 Hz, 1H), 8.08 (dd, *J* = 8.1, 2.2 Hz, 1H), 7.68–7.61 (m, 1H), 7.52 ppm (ddt, *J* = 9.4, 5.8, 3.0 Hz, 3H). ^13^C NMR (101 MHz, DMSO-*d*_6_): δ = 165.9, 149.4, 147.5, 138.1, 137.3, 135.7, 131.7, 131.5, 130.5, 130.0, 127.9, 124.2 ppm.

#### 5-(3-Chlorophenyl)picolinic Acid (**16c**)

Compound **16c** was prepared using 5-bromopicolinic acid (1 mmol, 202 mg) and 3-chlorophenyl boronic acid (1.3 eq, 203 mg) according to General Procedure C, affording compound **16c** as an off-pale pink solid (145 mg, 62%); HPLC – *t*_R_ = 3.938 min, >99% purity; LRMS [M + H]^+^ 234.0 *m*/*z*; ^1^H NMR (400 MHz, DMSO-*d*_6_): δ = ; 9.05 (d, *J* = 2.2 Hz, 1H), 8.31 (dd, *J* = 8.2, 2.4 Hz, 1H), 8.11 (d, *J* = 8.1 Hz, 1H), 7.92 (d, *J* = 2.0 Hz, 1H), 7.79 (dt, *J* = 7.1, 1.9 Hz, 1H), 7.61–7.51 ppm (m, 2H). ^13^C NMR (101 MHz, DMSO-*d*_6_): δ = 165.9, 147.7, 147.6, 138.23, 136.9, 135.5, 134.0, 131.1, 128.7, 126.9, 125.9, 124.8 ppm.

#### 5-(4-Chlorophenyl)picolinic Acid (**16d**)

Compound **16d** was prepared using 5-bromopicolinic acid (1 mmol, 202 mg) and 4-chlorophenyl boronic acid (1.3 eq, 203 mg) according to General Procedure C, affording compound **16d** as a white solid (130 mg, 56%); HPLC – *t*_R_ = 3.919 min, >85% purity; LRMS [M + H]^+^ 234.0 *m*/*z*; ^1^H NMR (401 MHz, DMSO-*d*_6_): δ 9.03 (dd, *J* = 2.4, 0.8 Hz, 1H), 8.28 (dd, *J* = 8.2, 2.4 Hz, 1H), 8.11 (dd, *J* = 8.2, 0.8 Hz, 1H), 7.89–7.83 (m, 2H), 7.64–7.58 (m, 2H). ^13^C NMR (101 MHz, DMSO-*d*_6_): δ 165.9, 147.5, 147.4, 137.1, 135.1, 134.9, 133.9, 129.2 (2C), 129.0 (2C), 124.8.

#### 5-(2-Methoxyphenyl)picolinic acid (**16g**)

Compound **16g** was prepared using 5-bromopicolinic acid (1 mmol, 202 mg) and 2-methoxyphenyl boronic acid (1.3 eq, 197 mg) according to General Procedure C, affording compound **16g** as a white solid (145 mg, 63%); HPLC – *t*_R_ = 3.395 min, >99% purity; LRMS [M + H]^+^ 230.1 *m*/*z*; ^1^H NMR (401 MHz, DMSO-*d*_6_): δ 8.80 (d, *J* = 1.6 Hz, 1H), 8.08 (d, *J* = 1.5 Hz, 2H), 7.49–7.40 (m, 2H), 7.21–7.16 (m, 1H), 7.10 (td, *J* = 7.4, 1.0 Hz, 1H), 3.80 ppm (s, 3H). ^13^C NMR (101 MHz, DMSO-*d*_6_): δ 166.0, 156.3, 149.5, 146.4, 137.6, 136.8, 130.1, 130.4, 125.3, 124.2, 121.1, 111.9, 55.7 ppm.

#### 5-(3-Methoxyphenyl)picolinic Acid (**16h**)

Compound **16h** was prepared using 5-bromopicolinic acid (1 mmol, 202 mg) and 3-methoxyphenyl boronic acid (1.3 eq, 197 mg) according to General Procedure C, affording compound **16h** as a white solid (166 mg, 74%); HPLC – *t*_R_ = 3.498 min, >99% purity; LRMS [M + H]^+^ 230.1 *m*/*z*; ^1^H NMR (401 MHz, DMSO-*d*_6_): δ 9.03 (dd, *J* = 2.3, 0.8 Hz, 1H), 8.27 (dd, *J* = 8.2, 2.4 Hz, 1H), 8.11 (dd, *J* = 8.1, 0.8 Hz, 1H), 7.46 (t, *J* = 7.9 Hz, 1H), 7.41–7.33 (m, 2H), 7.05 ppm (ddd, *J* = 8.2, 2.5, 1.0 Hz, 1H); ^13^C NMR (101 MHz, DMSO-*d*_6_): δ 166.02, 159.93, 147.62, 147.20, 138.21, 137.50, 135.25, 130.39, 124.76, 119.47, 114.57, 112.66, 55.30 ppm.

#### 5-(4-Methoxyphenyl)picolinic Acid (**16i**)

Compound **16i** was prepared using 5-bromopicolinic acid (1 mmol, 202 mg) and 4-methoxyphenyl boronic acid (1.3 eq, 197 mg) according to General Procedure C, affording compound **16i** as a white solid (170 mg, 75%); HPLC – *t*_R_ = 3.394 min, >90% purity; LRMS [M + H]^+^ 230.1 *m*/*z*; ^1^H NMR (400 MHz, DMSO-*d*_6_): δ 8.98 (d, *J* = 2.3 Hz, 1H), 8.21 (dd, *J* = 8.2, 2.4 Hz, 1H), 8.07 (d, *J* = 8.2 Hz, 1H), 7.78 (d, *J* = 8.8 Hz, 1H), 7.10 (d, *J* = 8.8 Hz, 2H), 3.82 ppm (s, 3H); ^13^C NMR (101 MHz, DMSO-*d*_6_): δ 166.1, 160.1, 146.9, 146.3, 138.0, 135.9, 134.3, 128.5, 128.2, 114.8, 112.9, 55.3 ppm.

#### 5-(*o*-Tolyl)picolinic Acid (**16j**)

Compound **16j** was prepared using 5-bromopicolinic acid (1 mmol, 202 mg) and 2-methylphenyl boronic acid (1.3 eq, 176 mg) according to General Procedure C, affording compound **16j** as a white solid (132 mg, 62%); HPLC – *t*_R_ = 3.544 min, >95% purity; LRMS [M + H]^+^ 214.1 *m*/*z*; ^1^H NMR (401 MHz, DMSO-*d*_6_): δ = 8.69 (dd, *J* = 2.2, 0.8 Hz, 1H), 8.11 (dd, *J* = 8.0, 0.8 Hz, 1H), 7.99 (dd, *J* = 8.0, 2.3 Hz, 1H), 7.39–7.28 (m, 4H), 2.26 ppm (s, 3H); ^13^C NMR (101 MHz, DMSO-*d*_6_): δ 166.1, 149.3, 146.8, 139.8, 137.6, 136.9, 135.2, 130.7, 129.7, 128.5, 126.3, 124.2, 19.9 ppm.

#### 5-(*m*-Tolyl)picolinic Acid (**16k**)

Compound **16k** was prepared using 5-bromopicolinic acid (1 mmol, 202 mg) and 3-methylphenyl boronic acid (1.3 eq, 176 mg) according to General Procedure C, affording compound **16k** as a white solid (156 mg, 73%); HPLC – *t*_R_ = 3.712 min, >95% purity; LRMS [M + H]^+^ 214.1 *m*/*z*; ^1^H NMR (401 MHz, DMSO-*d*_6_): δ 9.02–8.99 (m, 1H), 8.24 (dd, *J* = 8.2, 2.3 Hz, ^1^H), 8.11 (dd, *J* = 8.2, 0.8 Hz, 1H), 7.65–7.57 (m, 2H), 7.43 (t, *J* = 7.6 Hz, 1H), 7.29 (d, *J* = 7.5 Hz, 1H), 2.40 ppm (s, 3H); ^13^C NMR (101 MHz, DMSO-*d*_6_): δ 166.0, 147.5, 147.0138.6, 138.4, 136.0, 135.1, 129.5, 129.2, 127.8, 124.8, 124.3, 21.0 ppm.

#### 5-(*p*-Tolyl)picolinic acid (**16L**)

Compound **16L** was prepared using 5-bromopicolinic acid (1 mmol, 202 mg) and 4-methylphenyl boronic acid (1.3 eq, 176 mg) according to General Procedure C, affording compound **16L** as a white solid (120 mg, 56%); HPLC – *t*_R_ = 3.724 min, >95% purity; LRMS [M + H]^+^ 214.1 *m*/*z*; ^1^H NMR (401 MHz, DMSO-*d*_6_): δ 9.00 (d, *J* = 2.3 Hz, 1H), 8.22 (dd, *J* = 8.1, 2.3 Hz, 1H), 8.09 (d, *J* = 8.1 Hz, 1H), 7.70 (d, *J* = 7.8 Hz, 2H), 7.34 (d, *J* = 7.8 Hz, 2H), 2.36 ppm (s, 3H); ^13^C NMR (101 MHz, DMSO-*d*_6_): δ 166.0, 147.2, 146.8, 138.5, 138.2, 134.7, 133.1, 129.8, 127.0, 124.8, 20.7 ppm.

#### 5-(2-Cyanophenyl)picolinic Acid (**16e**)

Compound **16e** was prepared using 5-bromopicolinic acid (1 mmol, 202 mg) and 2-cyanophenyl boronic acid, piconol ester (1.3 eq, 297 mg) according to General Procedure C, affording the Compound **16e** as a pink solid (148 mg, 66%).

#### 5-(3-Cyanophenyl)picolinic Acid (**16f**)

Compound **16f** was prepared using 5-bromopicolinic acid (1 mmol, 202 mg) and 3-cyanophenyl boronic acid (1.3 eq, 191 mg) according to General Procedure C, affording compound **16f** as a brown solid (104 mg, 45%); HPLC – *t*_R_ = 3.261 min, >85% purity; LRMS [M + H]^+^ 225.1 *m*/*z*.

#### 5-(4-Cyanophenyl)picolinic Acid (**16g**)

Compound **16g** was prepared using 5-bromopicolinic acid (1 mmol, 202 mg) and 4-cyanophenyl boronic acid, pinacol ester (1.3 eq, 297 mg) according to General Procedure C, affording compound **16g** as an off-white solid (109 mg, 48%); HPLC – *t*_R_ = 3.724 min, >85% purity; LRMS [M + H]^+^ 225.1 *m*/*z*.

#### *N*′-(2-(1-Methyl-1*H*-benzo[d]imidazole-2-yl)acetyl)-2-naphthohydrazide (**13a**)

Compound **13a** was obtained using 2-(1-methyl-1*H*-benzo[*d*]imidazole-2-yl)acetohydrazide (60 mg, 0.3 mmol) and 2-naphthoic acid (35 mg, 0.2 mmol) following General Procedure B, affording compound **13a** as a white solid (55 mg 77%). HPLC – *t*_R_ = 4.217 > 98% purity at 254 nm; HRMS (ESI) [M + H]^+^ 358.1431 *m*/*z*; found 359.1504 *m*/*z*; 1H NMR (400 MHz, DMSO-*d*_6_): δ 10.62 (s, 1H), 10.45 (s, 1H), 8.53–8.48 (m, 1H), 8.09–7.90 (m, 4H), 7.70–7.50 (m, 4H), 7.21 (dtd, *J* = 22.3, 7.3, 1.3 Hz, 2H), 4.05 (s, 2H), 3.85 ppm (s, 3H).^13^C NMR (101 MHz, DMSO-*d*_6_): δ = 166.6, 165.5, 149.5, 142.0, 135.9, 134.3, 131.9, 129.6, 128.8, 128.1, 128.0, 127.9, 127.6, 126.8, 123.9, 121.7, 121.2, 118.4, 109.8, 33.2, 30.0 ppm.

#### *N*′-(2-(1-Methyl-1*H*-benzo[d]imidazole-2-yl)acetyl)picolinohydrazide (**13b**)

Compound **13b** was obtained using 2-(1-methyl-1*H*-benzo[*d*]imidazole-2-yl)acetohydrazide (60 mg, 0.3 mmol) and picolinic acid (25 mg, 0.2 mmol) following General Procedure A, affording compound **13b** as a white solid (46 mg, 74%). HPLC – *t*_R_ = 3.138, >99% purity at 254 nm; HRMS (ESI) [M + H]^+^ 310.1299 *m*/*z*; found 310.1302 *m*/*z*; ^1^H NMR (400 MHz, DMSO-*d*_6_): δ = 10.58 (s, 1H), 10.48 (s, 1H), 8.67 (dt, *J* = 4.8, 1.4 Hz, 1H), 8.11–7.93 (m, 2H), 7.67–7.62 (m, 1H), 7.60–7.49 (m, 2H), 7.20 (dtd, *J* = 22.1, 7.1, 1.3 Hz, 2H), 4.01 (s, 2H), 3.82 ppm (s, 2H); ^13^C NMR (101 MHz, DMSO-*d*_6_): δ = 166.1, 162.7, 149.4, 149.0, 148.6, 142.0, 137.8, 135.9, 126.9, 122.3, 121.8, 121.2, 118.4, 109.8, 33.2, 30.0 ppm.

#### *N*′-(2-(1-Methyl-1*H*-benzo[*d*]imidazole-2-yl)acetyl)benzohydrazide (**13c**)

Compound **13c** was obtained using 2-(1-methyl-1*H*-benzo[*d*]imidazole-2-yl)acetohydrazide (60 mg, 0.3 mmol) and benzoic acid (25 mg, 0.2 mmol) following General Procedure B, affording compound **13c** as a white solid (51 mg, 83%). HPLC – *t*_R_ = 3.334, >99% purity at 254 nm; HRMS (ESI) [M + H]^+^ 309.1346 *m*/*z*; found 309.1350 *m*/*z*; ^1^H NMR (400 MHz, DMSO-*d*_6_): δ = 10.42 (brs, 2H), 7.91–7.85 (m, 2H), 7.63–7.46 (m, 5H), 7.20 (dddd, *J* = 22.4, 8.3, 7.2, 1.2 Hz, 2H), 4.02 (s, 2H), 383 ppm (s, 3H); ^13^C NMR (101 MHz, DMSO-*d*_6_): δ = 166.6, 165.4, 149.4, 142.0, 135.9, 132.3, 131.8, 128.4, 127.4, 121.7, 121.3, 118.4, 109.9, 33.2, 30.0 ppm.

#### *N*′-(2-(1-Methyl-1*H*-benzo[*d*]imidazole-2-yl)acetyl)-6-phenylpicolinohydrazide (**13d**)

Compound **13d** was obtained using 2-(1-methyl-1*H*-benzo[*d*]imidazole-2-yl)acetohydrazide (60 mg, 0.3 mmol) and 6-phenylpyridine 2-carboxylic acid (40 mg, 0.2 mmol) following General Procedure A, affording Compound **13d** as a white solid (14 mg, 12%). HPLC – *t*_R_ = 4.538, >99% purity at 254 nm; LRMS [M + H]^+^ 386.2 *m*/*z*; HRMS (ESI) [M + H]^+^ 386.1612 *m*/*z*; found 386.1620 *m*/*z*; ^1^H NMR (400 MHz, DMSO-*d*_6_): δ = 10.70 (s, 1H), 10.51 (s, 1H), 8.44–8.32 (m, 2H), 8.23 (d, *J* = 7.9 Hz, 1H), 8.09 (td, *J* = 7.8, 1.5 Hz, 1H), 7.98 (d, *J* = 7.9 Hz, 1H), 7.62–7.45 (m, 5H), 7.28–7.15 (m, 2H), 4.06 (s, 2H), 3.85 ppm (s, 3H); ^13^C NMR (101 MHz, DMSO-*d*_6_): δ = 166.6, 162.8, 155.1, 149.5, 148.8, 141.9, 138.8, 137.2, 135.9, 129.6, 128.6, 127.1, 123.0, 121.8, 121.2, 120.9, 118.4, 109.8, 33.3, 30.0 ppm.

#### *N*′-(2-(1-Methyl-1*H*-benzo[d]imidazole-2-yl)acetyl)-5-phenylpicolinohydrazide (**13e**)

Compound **13e** was obtained using 2-(1-methyl-1*H*-benzo[*d*]imidazole-2-yl)acetohydrazide (80 mg, 0.4 mmol) and 5-phenylpyridine 2-carboxylic acid (60 mg, 0.3 mmol) following General Procedure B, affording the **13e** as a white solid (26 mg, 22%). HPLC – *t*_R_ = 4.474 min, >99% purity at 254 nm; HRMS (ESI) [M + H]^+^ 386.1612 *m*/*z*; found 386.1619 *m*/*z*; ^1^H NMR (400 MHz, DMSO-*d*_6_): δ = 10.64 (s, 1H), 10.52 (s, 1H), 8.98 (dd, *J* = 2.3, 0.8 Hz, 1H), 8.30 (dd, *J* = 8.2, 2.3 Hz, 1H), 8.10 (dd, *J* = 8.2, 0.8 Hz, 1H), 7.85–7.80 (m, 2H), 7.60–7.57 (m, 1H), 7.57–7.52 (m, 3H), 7.51–7.46 (m, 1H), 7.28–7.17 (m, 2H), 4.04 (s, 2H), 3.84 ppm (s, 3H); ^13^C NMR (101 MHz, DMSO-*d*_6_): δ = 166.1, 162.1, 149.4, 147.8, 146.7, 141.7, 138.3, 136.1, 135.9, 135.5, 129.2, 128.8, 127.2, 122.5, 121.9, 121.4, 118.4, 109.9, 33.2, 30.0 ppm.

#### 5-(2-Chlorophenyl)-*N*′-(2-(1-methyl-1*H*-benzo[d]imidazole-2-yl)acetyl)picolinohydrazide (**17a**)

Compound **17a** was obtained using 2-(1-methyl-1*H*-benzo[*d*]imidazole-2-yl)acetohydrazide (130 mg, 0.6 mmol) and 5-(2-chloropehnyl)picolinic acid (100 mg, 0.4 mmol) following General Procedure B, affording compound **17a** as a white solid (43 mg, 24%) HPLC – *t*_R_ = 4.564 min, >99% purity at 254 nm; HRMS (ESI) [M + H]^+^ 420.1222 *m*/*z*; found 420.1236 *m*/*z*; ^1^H NMR (400 MHz, DMSO-*d*_6_): δ = ; 10.70 (s, 1H), 10.53 (s, 1H), 8.74 (dd, *J* = 1.9, 1.1 Hz, 1H), 8.12 (t, *J* = 1.6 Hz, 2H), 7.68–7.62 (m, 1H), 7.54 (dddd, *J* = 19.4, 9.4, 6.7, 2.3 Hz, 5H), 7.26–7.14 (m, 2H), 4.03 (s, 2H), 3.83 ppm (s, 3H); ^13^C NMR (101 MHz, DMSO-*d*_6_): δ = 166.2162.6, 149.5, 148.6, 148.1, 142.9, 138.5, 137.3, 135.9, 135.7, 131.7, 131.5, 130.5, 130.0, 127.9, 121.9, 121.8, 121.3, 118.5, 109.9, 33.2, 30.1 ppm.

#### 5-(3-Chlorophenyl)-*N*′-(2-(1-methyl-1*H*-benzo[d]imidazole-2-yl)acetyl)picolinohydrazide (**17b**)

Compound **17b** was obtained using 2-(1-methyl-1*H*-benzo[*d*]imidazole-2-yl)acetohydrazide (130 mg, 0.6 mmol) and 5-(3-imidazole-2-yl)picolinic acid (100 mg, 0.4 mmol) following General Procedure B, affording compound **17b** as a white solid (46 mg, 26%) HPLC – *t*_R_ = 4.674 min, >99% purity at 254 nm; HRMS (ESI) [M + H]^+^ 420.1222 m/z; found 420.1242 *m*/*z*; ^1^H NMR (400 MHz, DMSO-*d*_6_): δ = ; 10.68 (s, 1H), 10.54 (s, 1H), 9.03 (dd, *J* = 20.1, 2.2 Hz, 1H), 8.36 (ddd, *J* = 12.1, 8.2, 2.4 Hz, 1H), 8.12 (dd, *J* = 15.0, 8.2 Hz, 1H), 7.94 (dt, *J* = 12.3, 1.8 Hz, 1H), 7.82 (ddt, *J* = 12.9, 7.1, 1.7 Hz, 1H), 7.61–7.52 (m, 4H), 7.22 (dtd, *J* = 21.8, 7.3, 1.2 Hz, 2H), 4.05 (s, 2H), 3.84 ppm (s, 3H); ^13^C NMR (101 MHz, DMSO-*d*_6_): δ = 166.1, 162.6, 149.4, 148.3, 146.9, 141.5, 138.3, 136.9, 135.9, 135.8, 134.0, 131.0, 128.7, 127.0, 126.0, 122.5, 122.0, 121.5, 118.3, 110.0, 33.2, 30.1 ppm.

#### 5-(4-Chlorophenyl)-*N*′-(2-(1-methyl-1*H*-benzo[*d*]imidazole-2-yl)acetyl)picolinohydrazide (**15c**)

Compound **15c** was obtained using 2-(1-methyl-1*H*-benzo[*d*]imidazole-2-yl)acetohydrazide (130 mg, 0.6 mmol) and 5-(4-imidazole-2yl)picolinic acid (100 mg, 0.4 mmol) following General Procedure B, affording compound **15c** as a white solid (73 mg, 42%); HPLC – *t*_R_ 4.706 min, >95% purity at 254 nm; HRMS (ESI) [M + H]^+^ 420.1222 *m*/*z*; found 420.124 *m*/*z*; ^1^H NMR (400 MHz, DMSO-*d*_6_): δ = 10.66 (s, 1H), 10.51 (s, 1H), 8.99 (dd, *J* = 2.4, 0.8 Hz, 1H), 8.32 (dd, *J* = 8.2, 2.3 Hz, 1H), 8.10 (dd, *J* = 8.2, 0.8 Hz, 1H), 7.89–7.85 (m, 2H), 7.65–7.49 (m, 4H), 7.27–7.14 (m, 2H), 4.02 (s, 2H), 3.83 ppm (s, 3H); ^13^C NMR (101 MHz, DMSO-*d*_6_): δ = 166.2, 162.6, 149.5, 148.0, 146.7, 142.0, 137.1, 135.9, 135.6, 134.9, 133.8, 129.2, 129.0, 122.5, 121.8, 121.63, 118.5, 109.9, 33.2, 30.0 ppm.

#### 5-(3-Cyanophenyl)-*N*′-(2-(1-methyl-1*H*-benzo[d]imidazole-2-yl)acetyl)picolinohydrazide (**17d**)

Compound **17d** was obtained using 2-(1-methyl-1*H*-benzo[*d*]imidazole-2-yl)acetohydrazide (130 mg, 0.6 mmol) and 5-(3-cyanophenyl)picolinic acid (100 mg, 0.4 mmol) following General Procedure B, affording compound **17d** as a off-white solid (15 mg, 8%); HPLC – *t*_R_ 4.098 min, >85% purity at 254 nm; HRMS (ESI) [M + H]^+^ 411.1564 *m*/*z*; found 411.1579 *m*/*z*; ^1^H NMR (400 MHz, DMSO-*d*_6_): δ = ; 10.69 (d, *J* = 10.3 Hz, 1H), 10.52 (s, 1H), 9.05 (dd, *J* = 2.3, 0.8 Hz, 1H), 8.40 (dd, *J* = 8.2, 2.3 Hz, 1H), 8.14 (dd, *J* = 8.2, 0.8 Hz, 1H), 8.07–7.99 (m, 4H), 7.60–7.51 (m, 2H), 7.27–7.13 (m, 2H), 4.03 (s, 2H), 3.83 ppm (s, 3H); ^13^C NMR (101 MHz, DMSO-*d*_6_): δ = 166.2, 162.5, 149.5, 148.7, 142.0, 140.6, 140.5, 136.6, 136.2, 135.9, 133.0, 128.2, 124.2, 122.5, 121.8, 121.3, 188.6, 111.4, 109.9, 33.2, 30.0 ppm.

#### 5-(4-Cyanophenyl)-*N*′-(2-(1-methyl-1*H*-benzo[d]imidazole-2-yl)acetyl)picolinohydrazide (**17e**)

Compound **17e** was obtained using 2-(1-methyl-1*H*-benzo[*d*]imidazole-2-yl)acetohydrazide (100 mg, 0.45 mmol) and 5-(4-cyanophenyl)picolinic acid (80 mg, 0.3 mmol) following General Procedure B, affording compound **17e** as an off-white solid (27 mg, 19%); HPLC – *t*_R_ = 4.098 min, >99% purity at 254 nm; HRMS (ESI) [M + H]^+^ 411.1564 *m*/*z*; found 411.158 *m*/*z*; ^1^H NMR (400 MHz, DMSO-*d*_6_): δ = ; 10.72 (s, 1H), 10.55 (s, 1H), 9.05 (d, *J* = 2.3 Hz, 1H), 8.40 (dd, *J* = 8.2, 2.3 Hz, 1H), 8.14 (d, *J* = 8.2 Hz, 1H), 8.04 (q, *J* = 8.4 Hz, 4H), 7.62–7.55 (m, 2H), 7.30–7.18 (m, 3H), 4.06 (s, 2H), 3.85 ppm (s, 3H); ^13^C NMR (101 MHz, DMSO-*d*_6_): δ = 166.0, 162.5, 148.7, 147.2, 140.9, 140.7, 136.6, 136.3, 135.6, 133.0, 128.2, 122.6, 122.3, 122.2, 118.6, 118.1, 111.4, 110.2, 33.0, 30.2, 23.4 ppm.

#### 5-(2-Methoxyphenyl)-*N*′-(2-(1-methyl-1*H*-benzo[d]imidazole-2-yl)acetyl)picolinohydrazide (**17f**)

Compound **17f** was obtained using 2-(1-methyl-1*H*-benzo[*d*]imidazole-2-yl)acetohydrazide (130 mg, 0.6 mmol) and 5-(2-methoxyphenyl)picolinic acid (100 mg, 0.4 mmol) following General Procedure B, affording compound **17f** as an off-white solid (37 mg, 22%); HPLC – *t*_R_ = 4.420 min, >99% purity; HRMS (ESI) [M + H]^+^ 416.1717 *m*/*z*; found 416.1736 *m*/*z*; ^1^H NMR (400 MHz, DMSO-*d*_6_): δ = ; 10.62 (s, 1H), 10.51 (s, 1H), 8.77 (dd, *J* = 2.1, 0.9 Hz, 1H), 8.17–8.05 (m, 2H), 7.60–7.50 (m, 2H), 7.49–7.41 (m, 2H), 7.27–7.15 (m, 3H), 7.10 (td, *J* = 7.4, 1.1 Hz, 1H), 4.02 (s, 2H), 3.83 (s, 3H), 3.80 ppm (s, 3H); ^13^C NMR (101 MHz, DMSO-*d*_6_): δ = 166.2, 162.7, 158.3., 149.5, 148.7, 147.2, 141.9, 138.9, 136.8, 135.9, 130.5, 130.4, 125.4, 121.9, 121.8, 121.36, 121.1, 118.5, 111.9, 109.96, 55.7, 33.2, 30.0 ppm.

#### 5-(3-Methoxyphenyl)-*N*′-(2-(1-methyl-1*H*-benzo[*d*]imidazole-2-yl)acetyl)picolinohydrazide (**17g**)

Compound **17g** was obtained using 2-(1-methyl-1*H*-benzo[*d*]imidazole-2-yl)acetohydrazide (130 mg, 0.6 mmol) and 5-(3-methoxyphenyl)picolinic acid (100 mg, 0.4 mmol) following General Procedure B, affording compound **17g** as an off-white solid (42 mg, 24%); HPLC – *t*_R_ 4.391 min, >99% purity; HRMS (ESI) [M + H]^+^ 416.1717 *m*/*z*; found 416.1734 *m*/*z*; ^1^H NMR (400 MHz, DMSO-*d*_6_): δ = ; 10.63 (s, 1H), 10.51 (s, 1H), 8.98 (d, *J* = 2.2 Hz, 1H), 8.31 (dd, *J* = 8.2, 2.3 Hz, 1H), 8.09 (d, *J* = 8.2 Hz, 1H), 7.55 (dd, *J* = 16.0, 7.9 Hz, 2H), 7.46 (t, *J* = 7.9 Hz, 1H), 7.40–7.33 (m, 2H), 7.27–7.14 (m, 2H), 7.09–7.01 (m, 1H), 4.02 (s, 2H), 3.85 ppm (s, 6H); ^13^C NMR (101 MHz, DMSO-*d*_6_): δ 166.2, 164.9, 162.7, 161.9, 159.9, 149.5, 147.9, 146.8, 138.3, 137.5, 135.7, 130.4, 122.5, 121.9, 121.4, 119.5, 118.4, 114.6, 112.6, 109.9, 55.3, 33.2, 30.1 ppm.

#### 5-(4-Methoxyphenyl)-*N*′-(2-(1-methyl-1*H*-benzo[d]imidazole-2-yl)acetyl)picolinohydrazide (**17h**)

Compound **17h** was obtained using 2-(1-methyl-1*H*-benzo[*d*]imidazole-2-yl)acetohydrazide (130 mg, 0.6 mmol) and 5-(4-methoxyphenyl)picolinic acid (100 mg, 0.4 mmol) following General Procedure B, affording compound **17h** as a white solid (56 mg, 32%); HPLC– *t*_R_ 4.366 min, >99% purity; HRMS (ESI) [M + H]^+^ 416.1717 *m*/*z*; found 416.1734 *m*/*z*; ^1^H NMR (400 MHz, DMSO-*d*_6_): δ = ; 10.60 (s, 1H), 10.50 (s, 1H), 8.94 (dd, *J* = 2.3, 0.8 Hz, 1H), 8.25 (dd, *J* = 8.2, 2.3 Hz, 1H), 8.06 (dd, *J* = 8.2, 0.8 Hz, 1H), 7.82–7.75 (m, 2H), 7.61–7.50 (m, 2H), 7.21 (dtd, *J* = 22.4, 7.3, 1.3 Hz, 2H), 7.12–7.07 (m, 2H), 4.02 (s, 2H), 3.83 ppm (d, *J* = 3.5 Hz, 6H); ^13^C NMR (101 MHz, DMSO-*d*_6_): δ = 166.2, 162.7, 160.0, 149.5, 147.0, 146.1, 142.0, 138.0, 135.9, 134.7, 128.5, 128.2, 122.5, 121.8, 121.3, 118.5, 114.7, 109.9, 55.3, 33.2, 30.0 ppm.

#### *N*′-(2-(1-Methyl-1*H*-benzo[*d*]imidazole-2-yl)acetyl)-5-(o-tolyl)picolinohydrazide (**17i**)

Compound **17i** was obtained using 2-(1-methyl-1*H*-benzo[*d*]imidazole-2-yl)acetohydrazide (142 mg, 0.7 mmol) and 5-(*o*-tolyl)picolinic acid (100 mg, 0.4 mmol) following General Procedure B, affording compound **17i** as a white solid (55 mg, 30%); HPLC – *t*_R_ 4.531 min, >95% purity; HRMS (ESI) [M + H]^+^ 400.1768 *m*/*z*; found 400.1787 *m*/*z*; ^1^H NMR (400 MHz, DMSO-*d*_6_): δ = ; 10.67 (s, 1H), 10.51 (s, 1H), 8.65 (dd, *J* = 2.2, 0.9 Hz, 1H), 8.10 (dd, *J* = 8.1, 0.9 Hz, 1H), 8.02 (dd, *J* = 8.0, 2.2 Hz, 1H), 7.60–7.50 (m, 2H), 7.42–7.28 (m, 4H), 7.21 (dtd, *J* = 22.4, 7.3, 1.2 Hz, 2H), 4.02 (s, 2H), 3.83 (s, 3H), 2.26 ppm (s, 3H); ^13^C NMR (101 MHz, DMSO-*d*_6_): δ 166.3, 162.8, 149.5, 148.5, 147.5, 142.1, 139.8, 138.1, 136.9, 136.0, 135.3, 130.7, 129.8, 128.6, 126.4, 122.1, 121.9, 121.4, 118.5, 109.9, 33.3, 30.1, 20.0 ppm.

#### *N*′-(2-(1-Methyl-1*H*-benzo[*d*]imidazole-2-yl)acetyl)-5-(*m*-tolyl)picolinohydrazide (**17j**)

Compound **17j** was obtained using 2-(1-methyl-1*H*-benzo[*d*]imidazole-2-yl)acetohydrazide (142 mg, 0.7 mmol) and 5-(*m*-tolyl)picolinic acid (100 mg, 0.4 mmol) following General Procedure B, affording compound **17j** as a white solid (52 mg, 29%); HPLC – *t*_R_ 4.630 min, >99% purity; HRMS (ESI) [M + H]^+^ 400.1768 *m*/*z*; found 400.1787 *m*/*z*; ^1^H NMR (400 MHz, DMSO-*d*_6_): δ = ; 10.65 (s, 1H), 10.53 (s, 1H), 8.96 (d, *J* = 2.2 Hz, 1H), 8.29 (dd, *J* = 8.2, 2.3 Hz, 1H), 8.09 (d, *J* = 8.2 Hz, 1H), 7.64 (d, *J* = 1.8 Hz, 1H), 7.63–7.55 (m, 3H), 7.43 (t, *J* = 7.6 Hz, 1H), 7.32–7.19 (m, 3H), 4.06 (s, 2H), 3.85 (s, 3H), 2.40 ppm (s, 3H); ^13^C NMR (101 MHz, DMSO-*d*_6_): δ = 166.0, 162.7, 149.4, 147.7, 146.7, 138.6, 136.0, 135.5, 129.5, 129.2, 127.8, 124.3, 122.5, 110.2, 33.0, 30.2, 21.0 ppm.

#### *N*′-(2-(1-Methyl-1*H*-benzo[d]imidazole-2-yl)acetyl)-5-(*p*-tolyl)picolinohydrazide (**17k**)

Compound **17k** was obtained using 2-(1-methyl-1*H*-benzo[*d*]imidazole-2-yl)acetohydrazide (142 mg, 0.7 mmol) and 5-(*p*-tolyl)picolinic acid (100 mg, 0.4 mmol), following General Procedure B, affording compound **17k** as a white solid (70 mg, 38%); HPLC – *t*_R_ 4.592 min, >99% purity; HRMS (ESI) [M + H]^+^ 400.1768 *m*/*z*; found 400.1785 *m*/*z*; ^1^H NMR (400 MHz, DMSO-*d*_6_): δ = ; 10.60 (s, 2H), 8.95 (dd, *J* = 2.3, 0.8 Hz, 1H), 8.27 (dd, *J* = 8.2, 2.3 Hz, 1H), 8.08 (dd, *J* = 8.2, 0.8 Hz, 1H), 7.75–7.69 (m, 2H), 7.61–7.50 (m, 2H), 7.35 (d, *J* = 7.9 Hz, 2H), 7.21 (dtd, *J* = 22.3, 7.3, 1.3 Hz, 2H), 4.02 (s, 2H), 3.83 (s, 3H), 2.37 ppm (s, 3H); ^13^C NMR (101 MHz, DMSO-*d*_6_): δ = 166.2, 162.7, 149.5, 147.5, 146.5, 142.0, 138.5, 138.3, 136.0, 135.2, 133.2, 129.9, 127.0, 122.6, 121.8, 121.4, 118.5, 109.9, 33.3, 30.1, 20.8 ppm.

**Table 4:**
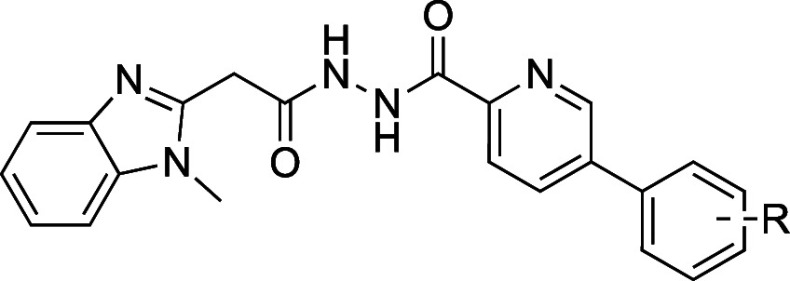

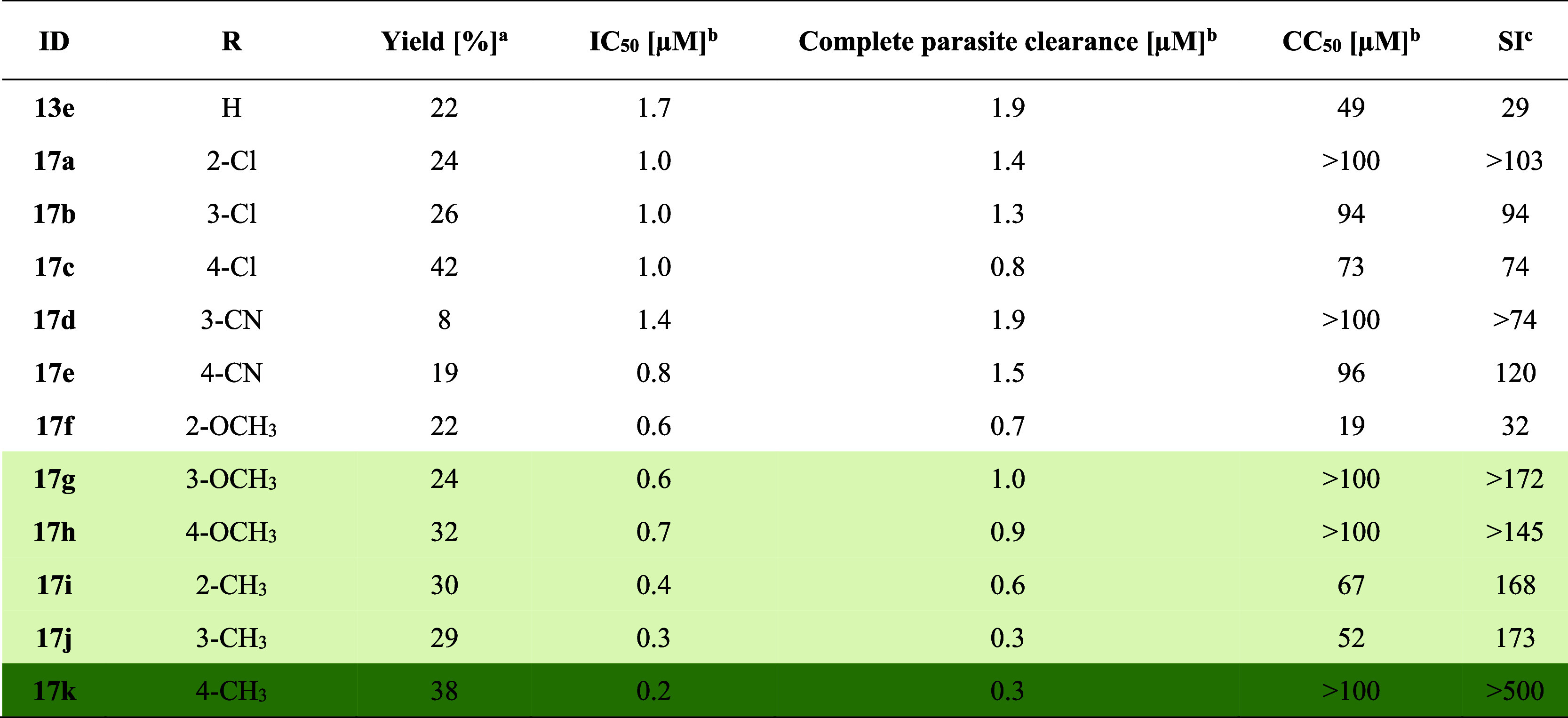
Structures, Anti *L. donovani* Activity, Cytotoxicity and SI with Right-Hand-Side Modification

a Yield refers to the final amide coupling step.

b Anti *L. donovani* activity and toxicity measured in THP-1 macrophage host cells using a top concentration of 100 μM (2× serial dilution 10-point curve). Experiments were performed in duplicates in one independent experiment, *n* = 1.

c SI—CC_50_/IC_50_. CC_50_—half-maximal cytotoxic concentration. IC_50_—half maximal inhibition concentration (reduction of total number of parasites by 50%). Highlighted in green: top lead compounds identified in this study (**17g–j**; light green); best performing compound (**17k**; dark green).

## Abbreviations

BSF: bloodstream form
CC_50_: half maximal cytotoxic concentration
CO_2_: carbon dioxide
Cl_int_: intrinsic clearance
CnBr: cyanogen bromide
DCM: dichloromethane
DIPA: diisopropylamine
DIPEA: *N*,*N*-diisopropylethylamine
DMF: dimethylformamide
DMSO: dimethyl sulfoxide
ESI: electrospray ionization
EtOAc: ethyl acetate
EtOH: ethanol
FBS: fetal bovine serum
FLINT: fluorescent intensity
HBTU: *O*-(benzotriazol-1-yl)*N*,*N*,*N*′,*N*′-tetramethyluronium; hexafluorophosphate
HCl: hydrochloric acid
HEPES: (4-(2-hydroxyethyl)-1-piperazineethanesulfonic acid)
HepG2: hepatoma G2, human liver cancer cell line
HPLC: high-performance liquid chromatography
HRMS: high-resolution mass spectrometry
IC_50_: half maximal inhibitory concentration
IR: infection ratio
K_2_CO_3_: potassium carbonate
*L. donovani*: *Leishmania donovani*
LCMS: liquid chromatography–mass spectrometry
LHS: left-hand side
LRMS: low-resolution mass spectrometry
MeOH: methanol
MgSO_4_: magnesium sulfate
*n*-BuLi: *n*-butyllithium
NaHCO_3_: sodium hydrogen carbonate
NaOH: sodium hydroxide
NMR: nuclear magnetic resonance
(*P*/φ): parasites per macrophage
Pd(PPh_3_)_4_: tetrakis(triphenylphosphine)palladium(0)
RHS: right-hand side
r.t.: room temperature
SAR: structure–activity relationship
SI: selectivity index
*t*_1/2_: half-life
THF: tetrahydrofuran
THP-1: human monocytic cell line derived from an acute monocytic leukemia patient
tR: retention time
VL: visceral leishmaniasis.
